# *In vivo* growth of *Staphylococcus lugdunensis* is facilitated by the concerted function of heme and non-heme iron acquisition mechanisms

**DOI:** 10.1016/j.jbc.2022.101823

**Published:** 2022-03-10

**Authors:** Ronald S. Flannagan, Jeremy R. Brozyna, Brijesh Kumar, Lea A. Adolf, Jeffrey John Power, Simon Heilbronner, David E. Heinrichs

**Affiliations:** 1 Department of Microbiology and Immunology, University of Western Ontario, London, Ontario, Canada; 2 Department of Infection Biology, Interfaculty Institute of Microbiology and Infection Medicine, University of Tübingen, Tübingen, Germany; 3 German Centre for Infection Research (DZIF), Partner Site Tübingen, Tübingen, Germany; 4 Cluster of Excellence EXC2124 Controlling Microbes to Fight Infections, Tübingen, Germany

**Keywords:** infection, iron, microbial pathogenesis, siderophore, heme, bacteria, aTc, anhydrotetracycline, CFU, colony-forming unit, CoNS, coagulase-negative staphylococci, C-TMS, TMS treated with 5% (w/v) Chelex-100 resin, DHBA, 2,3-dihydroxybenzoic acid, DFO, deferoxamine, ECF, energy coupling factor, EDDHA, ethylenediamine-di(o-hydroxyphenylacetic acid), Fhu, ferric hydroxamate uptake, Fur, ferric iron uptake repressor, HS, horse serum, IPTG, isopropyl-β-D-1-thiogalactopyranoside, Isd, iron-regulated surface determinant, L-DOPA, L-3,4-dihydroxyphenylalanine, LB, Luria-Bertani, NE, norepinephrine, RPMI-CAs, RPMI supplemented with casamino acids, SA, staphyloferrin A, SB, staphyloferrin B, TMS, Tris-minimal succinate, TSA, TSB solidified with 1.5% (w/v) agar, TSB, tryptic soy broth

## Abstract

*Staphylococcus lugdunensis* has increasingly been recognized as a pathogen that can cause serious infection indicating this bacterium overcomes host nutritional immunity. Despite this, there exists a significant knowledge gap regarding the iron acquisition mechanisms employed by *S. lugdunensis*, especially during infection of the mammalian host. Here we show that *S. lugdunensis* can usurp hydroxamate siderophores and staphyloferrin A and B from *Staphylococcus aureus*. These transport activities all required a functional FhuC ATPase. Moreover, we show that the acquisition of catechol siderophores and catecholamine stress hormones by *S. lugdunensis* required the presence of the *sst*-1 transporter-encoding locus, but not the *sst*-2 locus. Iron-dependent growth in acidic culture conditions necessitated the ferrous iron transport system encoded by *feoAB*. Heme iron was acquired *via* expression of the iron-regulated surface determinant (*isd*) locus. During systemic infection of mice, we demonstrated that while *S. lugdunensis* does not cause overt illness, it does colonize and proliferate to high numbers in the kidneys. By combining mutations in the various iron acquisition loci (*isd*, *fhuC*, *sst*-1, and *feo*), we demonstrate that only a strain deficient for all of these systems was attenuated in its ability to proliferate to high numbers in the murine kidney. We propose the concerted action of heme and non-heme iron acquisition systems also enable *S. lugdunensis* to cause human infection.

Iron (Fe) is an essential nutrient for nearly all forms of life, and despite its importance and abundance on earth, it primarily exists, at neutral pH, in an insoluble, ferric iron (Fe^3+^) state. In the context of the host, free Fe is scarce and is maintained at a concentration well below the requirement needed to support microbial growth (1). Within the host, most Fe is contained within heme prosthetic groups in hemoglobin inside circulating erythrocytes (2). Fe may also be sequestered within host cells by the iron-storage protein ferritin or bound by extracellular serum glycoproteins such as transferrin and lactoferrin (3). Collectively, these iron-binding proteins sequester Fe to minimize toxicity and to purpose this trace metal for host cellular processes. In addition, these proteins along with other immune effectors, including hepcidin (4, 5), ferroportin (4, 6), and calprotectin (7, 8), collectively act to limit Fe availability to invading microorganisms through a process termed nutritional immunity (9, 10). Therefore, to successfully colonize and infect a host, an invading bacterial pathogen must overcome host-driven nutrient sequestration and acquire Fe to support growth *in vivo* (1).

*Staphylococcus lugdunensis*, like several other coagulase-negative staphylococci (CoNS), is a human skin commensal and even protects against colonization by *Staphylococcus aureus* (11). However, it can also act as a pathogen that displays elevated virulence as compared to other CoNS (12, 13, 14, 15). Indeed, infections caused by *S. lugdunensis* are reminiscent of those attributed to *S. aureus*, and in a susceptible host, *S. lugdunensis* can cause a spectrum of infections including skin and soft tissue infections, bacteremia, pneumonia, and osteomyelitis (12, 13, 16, 17). In addition, *S. lugdunensis* has a propensity to cause aggressive infective endocarditis with mortality rates that can be as high as 50% (18, 19). The ability of *S. lugdunensis* to cause infection requires the concerted action of numerous virulence factors (20, 21) and necessitates that this bacterium acquires Fe from its host.

Successful pathogens can deploy a variety of mechanisms to acquire Fe from the host, and this can involve extracting Fe from both heme and non-heme Fe sources to support growth during infection (9, 22). Indeed, in Gram-positive bacteria such as *S. aureus*, the iron-regulated surface determinant (Isd) system functions to shuttle heme from the extracellular milieu across the bacterial cell wall and cytoplasmic membrane where Fe atoms can be extracted (22, 23, 24, 25, 26, 27). *S. lugdunensis* is unique among CoNS in that it also encodes a functional Isd system (28, 29, 30, 31) and utilizes this high affinity heme uptake pathway to grow at low (<500 nM) heme concentrations (32, 33, 34, 35). In addition, *S. lugdunensis* utilizes a high-affinity energy coupling factor (ECF) type transporter named Lha to extract heme from diverse host hemoproteins (36). To acquire non-heme Fe, most bacteria produce low molecular weight high-affinity iron chelators termed siderophores (37). Through siderophore production, bacteria can extract Fe^3+^ from oxyhydroxide precipitates or, for pathogens, from host glycoproteins such as transferrin; siderophore production has been shown in many bacteria including *S. aureus* to contribute significantly to pathogenesis *in vivo* (37). *S. aureus* elaborates two carboxylate-type siderophores, staphyloferrin A (SA) and staphyloferrin B (SB), of which the biosynthetic proteins are encoded by *sfa* and *sbn* loci, respectively (38, 39, 40). SA and SB are transported by the ABC transporters HtsABC and SirABC, respectively, which are encoded by loci adjacent to their cognate siderophore biosynthetic genes (41, 42). Contrary to *S. aureus*, *S. lugdunensis* does not produce either SA or SB (35); however, *S. lugdunensis* expresses the transporters HtsABC and SirABC and can thus usurp SA and SB produced by *S. aureus* (35). *S. aureus* can also utilize xenosiderophores (*i.e.*, siderophores produced by other microbes), and their utilization requires expression of the ferric hydroxamate uptake (Fhu) transporter and the Sst catechol transporter (43, 44). While Fhu enables *S. aureus* to utilize hydroxamate-type siderophores, Sst allows *S. aureus* to utilize siderophores containing catechol/catecholamine moieties (38, 43, 45). Host-derived stress hormones such as epinephrine and dopamine are catecholamines that chelate Fe and act as ‘pseudosiderophores’ to bacteria expressing catechol transport systems (44, 46, 47, 48, 49). Indeed, *S. aureus* utilizes the Sst pathway for catechol utilization, but Sst functionality is only evident when endogenous biosynthesis of SA and SB is perturbed (44).

In comparison to *S. aureus*, there exists a paucity of information regarding the Fe acquisition mechanisms employed by *S. lugdunensis*, especially during infection of the mammalian host. The lack of information on *S. lugdunensis* takes on added significance when one considers the pathogenic potential of this microbe. To rectify this, we investigated the iron procurement strategies of *S. lugdunensis* both *in vivo* and *in vitro*. We demonstrate that *S. lugdunensis* encodes and utilizes the Fhu and Sst transport proteins to acquire iron from a variety of hydroxamate and catechol/catecholamine siderophores and that the ferrous iron transport system, encoded by the *feoAB* genes, functions in *S. lugdunensis* to acquire iron under acidic culture conditions. During systemic infection of mice with *S. lugdunensis*, the bacteria seed the kidneys where they subsequently proliferate to high numbers. We demonstrate that growth of *S. lugdunensis* in the murine kidney requires the concerted action of heme (*i.e.*, Isd) and non-heme (including *feo*) iron acquisition systems.

## Results

### *S. lugdunensis* is restricted by serum *in vitro* yet the bacteria proliferate *in vivo*

**Figure 1:**
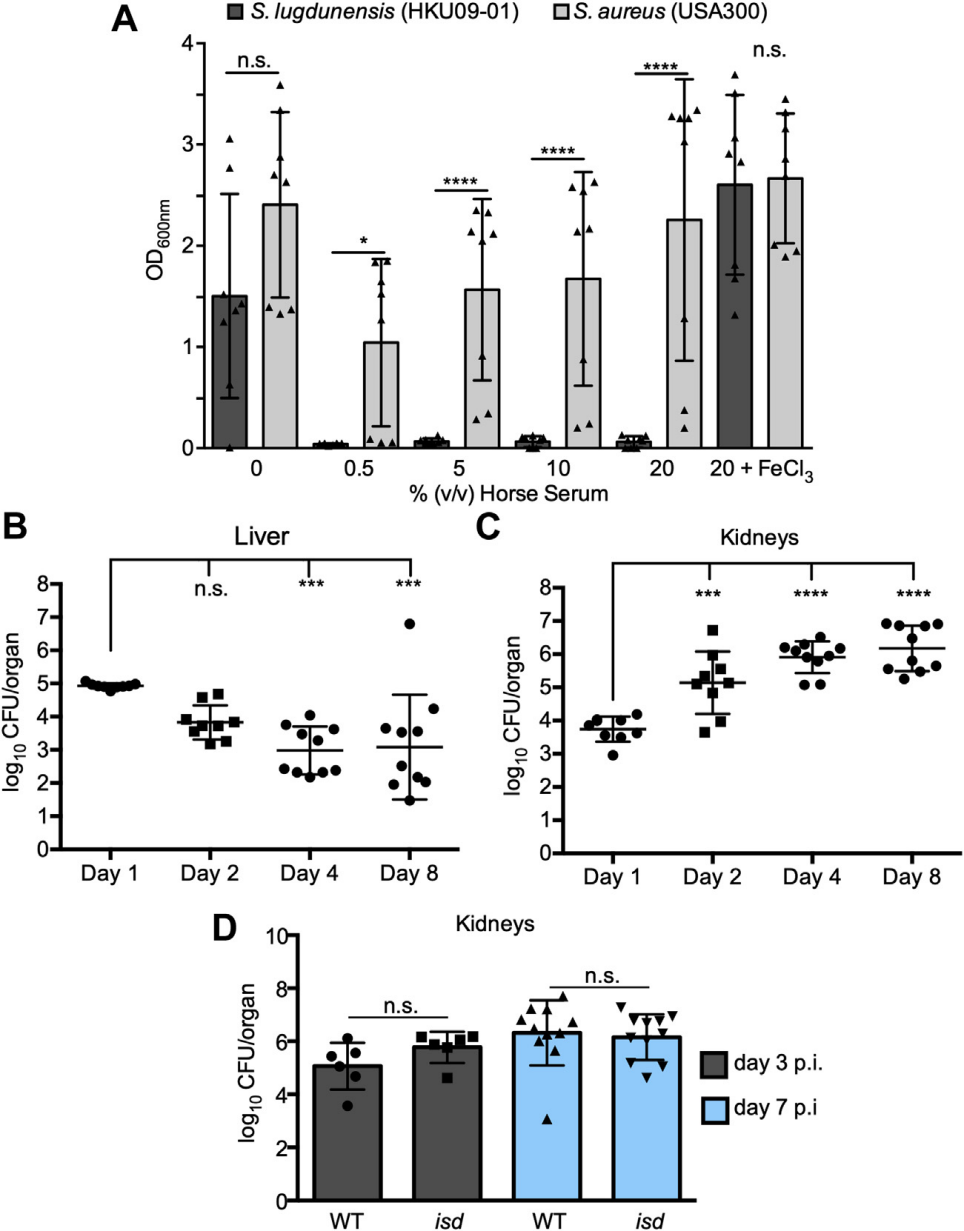
***S. lugdunensis* proliferates *in vivo* despite being restricted by serum.***A*, the growth of WT *S. lugdunensis* HKU09-01 and WT *S. aureus* USA300 in the presence of varying amounts of heat-inactivated horse serum in RPMI +1% (w/v) casamino acids is shown. The data presented are the mean ± standard deviation of the endpoint optical density at 600 nm (OD_600 nm_) measured after 24 h. The data derive from three independent experiments, and each symbol represents a separate biological replicate. Statistical significance was determined by performing an ordinary two-way ANOVA. *B* and *C*, the burden of *S. lugdunensis* HKU09-01 in the kidneys and liver, respectively, of infected mice at different days postinfection is shown. *C*, the burden of *S. lugdunensis* HKU09-01 and an *isd* mutant at day 3 and day 7 postinfection is shown. *B*–*D*, the data are presented as the mean log10 CFU/organ ± standard deviation, and each data point represents an infected animal. Statistical significance was measured by ordinary one-way ANOVA with a Tukey’s multiple comparison posttest. n.s. denotes not significant and ∗*p* < 0.05, ∗∗∗*p* < 0.001, ∗∗∗∗*p* < 0.0001. CFU, colony-forming unit; Isd, iron-regulated surface determinant.

Human infection by *S. lugdunensis* can be severe, and this coagulase-negative *Staphylococcus* spp. is often erroneously identified as *S. aureus*. Given that the ability to acquire iron from the host is essential for bacteria to cause infection, we speculated that *S. lugdunensis* and *S. aureus* may display similar capacity for iron acquisition. Remarkably, these two related yet distinct bacterial species demonstrate profound differences with respect to growth under conditions of iron restriction *in vitro* (Fig. 1*A*). Indeed, comparison of *S. aureus* USA300 and *S. lugdunensis* HKU09-01 growth in RPMI supplemented with casamino acids (CAs) and increasing amounts of horse serum (HS), a source of iron-chelating transferrin, revealed that *S. lugdunensis* failed to grow in as little as 0.5% (v/v) HS (Fig. 1*A*). The inability of *S. lugdunensis* to grow in the presence of HS was rescued upon supplementation of the culture medium with 20 μM FeCl_3_ establishing that the growth defect was due to iron restriction (Fig. 1*A*). In contrast, *S. aureus* USA300 grew robustly in 40× as much HS indicating these two bacterial species display profound differences in their ability to acquire iron *in vitro*. Despite this discrepancy, *S. lugdunensis* can cause serious infection, and therefore, we posited this bacterium must employ mechanisms of iron acquisition that permit growth *in vivo*. To test this notion, we next performed systemic murine infection experiments where the ability of *S. lugdunensis* HKU09-01 to colonize and grow in the kidneys and liver of infected animals was evaluated up to 8 days postinfection (Fig. 1, *B* and *C*). The murine kidney and liver were selected for this analysis as *S. lugdunensis* better colonizes these organs as compared to the heart and spleen (Fig. S1). These experiments revealed that in the murine liver, the burden of *S. lugdunensis* decreased over time, and by day 4 postinfection, an approximate 2-log reduction in bacterial burden was apparent (Fig. 1*B*). In contrast, in the murine kidney, it was evident that the burden of *S. lugdunensis* steadily increased over the 8-days infection where the bacterial counts increased by more than 2-log (Fig. 1*C*). These data indicate that *S. lugdunensis* HKU09-01 displays organ-specific differences in bacterial proliferation and indicate that within the kidney, *S. lugdunensis* must acquire iron within the murine host to support the significant bacterial growth.

### Isd-deficient *S. lugdunensis* HKU09-01 does not display growth defects within the murine kidney

That *S. lugdunensis* HKU09-01 grows within the murine kidney prompted us to investigate whether the high-affinity heme acquisition pathway encoded by the *isd* genes contributed to this phenotype. In *S. lugdunensis* HKU09-01, the entire *isd* locus is tandemly duplicated, and previous work characterizing Isd function in *S. lugdunensis* has demonstrated that an increased copy number of the *isd* locus enhances *S. lugdunensis* growth through heme utilization (35, 50). Moreover, it has been demonstrated that heme may be accessible to staphylococci within the murine kidney as compared to other organs which could ostensibly support *S. lugdunensis* growth (51). To evaluate the contribution of the *isd* genes to growth in the murine kidney, animals were infected with WT *S. lugdunensis* or an isogenic *isd* mutant where the tandemly duplicated *isd* loci have been deleted. Importantly, *in vitro* growth of this strain in the presence of hemin (ferric chloride heme) is impaired relative to WT (Fig. S2). *In vivo*, however, *isd*-deficient *S. lugdunensis* did not display a reduced bacterial burden in the murine kidney, as compared to the WT, at day 3 or day 7 postinfection indicating additional iron acquisition systems must help to support growth of *S. lugdunensis in vivo*.

### *S. lugdunensis* utilizes the fhu genes to acquire iron *in vitro*

**Figure 2:**
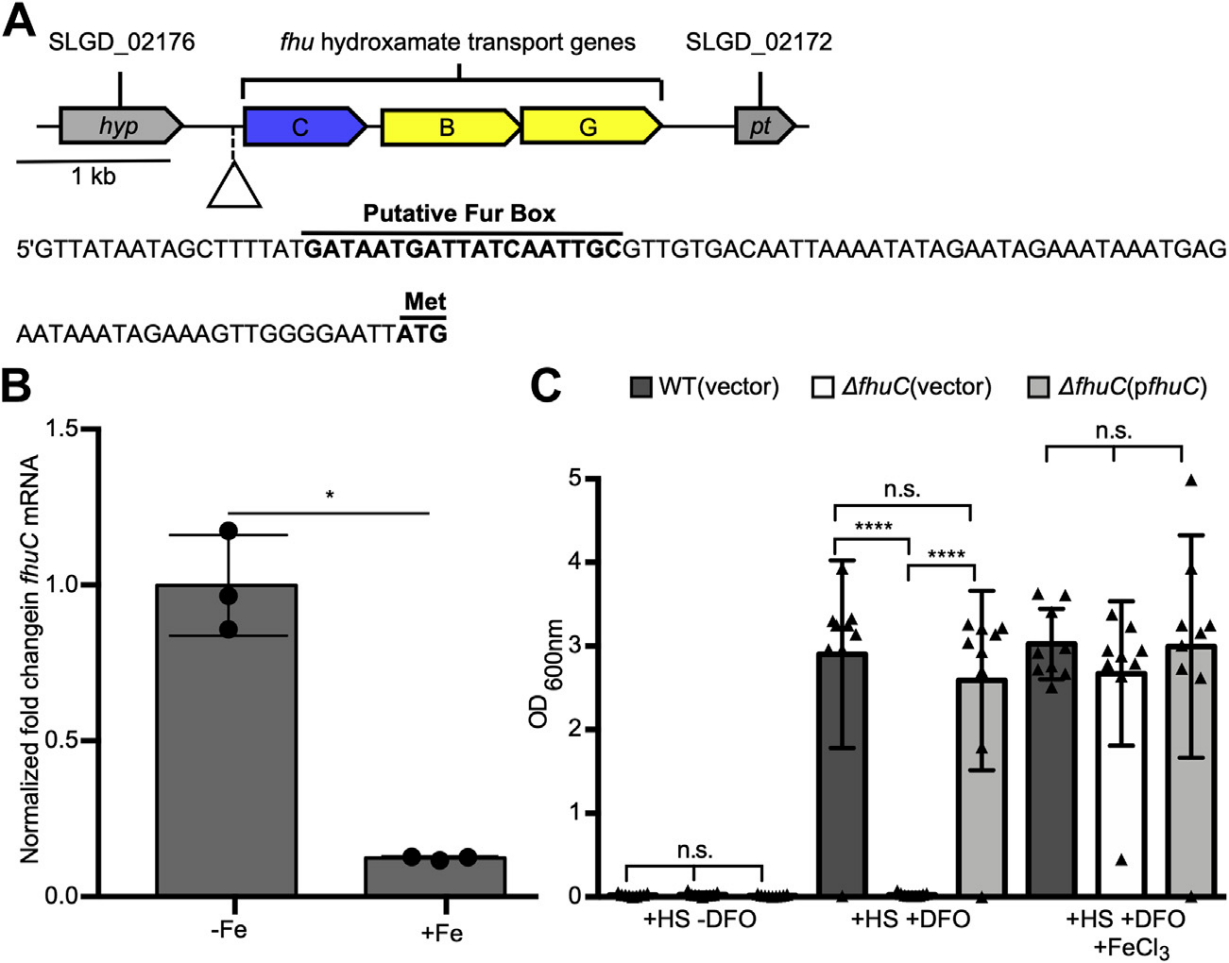
***Staphylococcus lugdunensis* deficient for th*e fhuC* gene fails to utilize deferoxamine (DFO) for growth under conditions of iron restriction.***A*, the physical map of the ferric hydroxamate uptake genes in *S. lugdunensis* is shown. The promoter sequence for the operon is shown, with the putative Fur box and start codon labeled. *B*, qPCR analysis of *fhuC* gene expression by WT *S. lugdunensis* grown in C-TMS (−Fe) or C-TMS with 100 μM FeCl_3_ (+Fe) is shown. Data were normalized relative to *rpoB* expression, and gene expression was normalized relative to that in C-TMS without added iron. The data derive from three independent experiments, and a total of three biological replicates and statistical significance was determined by Students *t* test. *C*, the growth of WT *S. lugdunensis* HKU09-01 and Δ*fhuC* with either the vector control or the p*fhuC* plasmid. Growth was in RPMI +1% (w/v) casamino acids with 0.05% (v/v) horse serum (HS) in the presence and absence of 100 μM DFO with or without 20 μM FeCl_3_. The data presented are the mean ± standard deviation of the endpoint optical density at 600 nm (OD_600 nm_) measured after 24 h. The data derive from three independent experiments, and each *symbol* represents a separate biological replicate. Statistical significance was determined by ordinary one-way ANOVA with a Tukey’s posttest. In (*B*) and (*C*) n.s. indicates not significant, ∗*p* < 0.05, ∗∗∗∗*p* < 0.0001. C-TMS, TMS treated with 5% (w/v) Chelex-100 resin; Fur, ferric iron uptake repressor.

Given *isd* is dispensable for *S. lugdunensis* growth within the murine kidney, we sought to identify the additional iron acquisition systems that operate in this bacterium. Searches of the available genome sequences of *S. lugdunensis* identified genes homologous to the *S. aureus fhuCBG* locus that, in *S. aureus*, are required for ferric hydroxamate siderophore-dependent iron acquisition (43). The proteins encoded by the *fhuCBG* genes in *S. lugdunensis* share significant identity to *fhuCBG* in *S. aureus*, and previous work has demonstrated that *fhuC*-deficient *S. aureus* is debilitated for growth in iron-deplete laboratory media and *in vivo* (45). This prompted us to assess the importance of *fhuC* during iron-restricted growth of *S. lugdunensis*. Analysis of the genomic sequence surrounding the *fhuCBG* locus in *S. lugdunensis* revealed that a canonical ferric iron uptake repressor (Fur) box lies upstream of the *fhuCBG* locus suggesting the Fur protein and cellular iron regulate transcription of these genes (Fig. 2*A*). In agreement with this notion, qPCR analysis revealed that the *fhu* genes in *S. lugdunensis* are significantly downregulated in response to Fe supplementation of the growth medium (Fig. 2*B*). This suggests that in low-iron environments, *S. lugdunensis* may utilize hydroxamate siderophores, if present, as a source of iron. To test this, we created an in-frame *fhuC* deletion in *S. lugdunensis* HKU09-01 to assess the role of *fhuC* in bacterial growth in the presence of deferoxamine (DFO) as a sole source of iron (Fig. 2*C*). Indeed, DFO can function as a xenosiderophore for *S. aureus* and chelate iron from transferrin owing to its exceptionally high affinity for Fe (52). This analysis revealed that *S. lugdunensis* lacking *fhuC* failed to utilize DFO for growth in the presence of HS, and this growth defect could be rescued by supplementation of the growth medium with FeCl_3_. Moreover, provision of *fhuC* encoded on a plasmid also restored DFO utilization to the *fhuC* mutant establishing the observed growth defect in *S. lugdunensis* was attributable to *fhuC* inactivation alone (Fig. 2*C*).

In *S. aureus*, the FhuC protein functions as a promiscuous ATPase that provides energy for the transport of hydroxamate type xenosiderophores through the Fhu system, as well as the carboxylate siderophores, SA and SB, that are transported into the cell through dedicated permeases encoded by the *htsABC* and *sirABC* operons, respectively (38, 41, 45, 53). In agreement with its predicted role in ferric hydroxamate uptake, the *fhuC* mutant was unable to use a variety of hydroxamate siderophores as an iron source in addition to DFO (Fig. S3). As expected, the *fhuC* mutant retained the ability to utilize catecholamine type siderophores and citrate (Fig. S3) that are transported through other dedicated siderophore uptake systems. Provision of the *fhuC* gene in *trans* eliminated the growth defects in the presence of hydroxamate siderophores confirming the importance of FhuC to xenosiderophore utilization. Of note, the *fhuC* mutant was also unable to utilize either SA or SB (*i.e.*, carboxylate siderophores) when supplied as a sole source of iron (Fig. S3) indicating that akin to *S. aureus*, *S. lugdunensis* utilizes the FhuC ATPase to energize uptake of SA and SB through HtsABC and SirABC, respectively (38, 45). Taken together, these data reveal that *S. lugdunensis* HKU09-01 is reliant on the *fhuC* gene to utilize SA and SB, in addition to hydroxamate type siderophores, for growth under iron limited conditions.

Given the promiscuity of FhuC in affecting uptake of various siderophores, we wished to investigate whether it had any role to play in heme uptake. Within the *isd* locus is a gene encoding an ATPase (*isdL*). Strain HKU09-01 has a duplicated *isd* locus (>30 kb duplication); so to investigate this, we used strain N920143 (single *isd* locus) to construct strains bearing mutations in *isdL* and *fhuC*. While the *isdL* mutant had a significant growth defect on hemoglobin as a sole iron source, the *fhuC* mutant did not, nor did it have any additive effect to *isdL* mutation on growth on hemoglobin as an iron source (Fig. S4*A*). Moreover, using bacterial two-hybrid analyses, we demonstrated that IsdL, and not FhuC, interacted with the IsdF membrane permease (Fig. S4*B*), providing further proof that the Isd heme acquisition system has a dedicated ATPase to power heme uptake, and that FhuC does not function in heme acquisition, rather it operates with the siderophore transporters in *S. lugdunensis*.

### In *S. lugdunensis*, the sst genes are required for catecholamine-dependent iron acquisition

**Figure 3:**
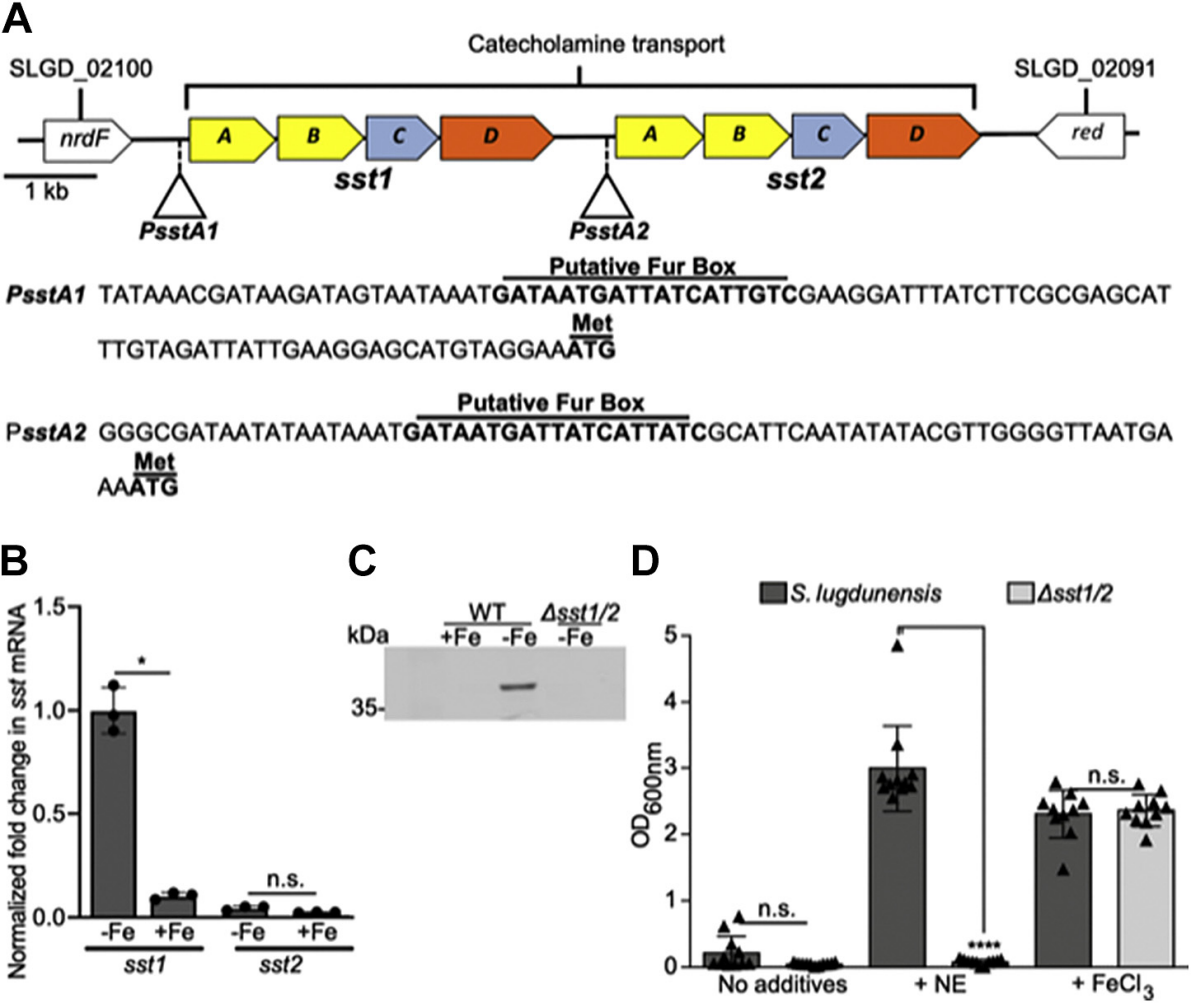
**The ability of *S. lugdunensis* to utilize catecholamines for growth under iron restriction requires the *sst* genes.***A*, a physical map of putative *sst*ABCD ferric–catecholamine acquisition genes in *S. lugdunensis* is shown. The promoter sequences for each gene set are shown, with putative Fur boxes and start codons labeled. In *S. lugdunensis*, the *sst* locus is duplicated giving rise to the operons labeled *sst*-1 and *sst*-2 respectively. *B*, the graph depicts qPCR analysis of *sst1* and *sst2* gene expression by *S. lugdunensis* grown overnight in C-TMS (−Fe) or C-TMS with 100 μM FeCl_3_ (+Fe). Data were normalized relative to *rpoB* expression, and expression was normalized relative to *sst1* in C-TMS without added iron (set as 1) as the comparator. The data derive from three independent experiments, and a total of three biological replicates and statistical significance was determined by Students *t* test. *C*, a representative Western blot demonstrating iron-regulated expression of SstD1 in WT *S. lugdunensis* and a Δ*sst*-1/2 mutant is shown. Cultures were grown overnight in C-TMS (−Fe) or C-TMS with addition of 100 μM FeCl_3_ (+Fe). Antisera raised against *S. aureus* SstD was used to immunodetect SstD (38 kDa) from *S. lugdunensis*. *D*, the ability of *sst*-1/2-deficient *S. lugdunensis* to utilize norepinephrine (NE) as an iron source as compared to WT is shown. The bacteria were grown in RPMI with 1% (w/v) casamino acids and 0.05% (v/v) heat-inactivated horse serum. NE was added at 50 μM, and FeCl_3_ was used as a control at 20 μM. The data shown are the mean ± standard deviation of the endpoint optical density at 600 nm (OD_600 nm_) measured after 24 h. The data derive from three independent experiments, and each symbol represents a separate biological replicate. Statistical significance was determined by ordinary one-way ANOVA with a Tukey’s posttest. In *B* and *D*, n.s. indicates not significant, ∗*p* < 0.05, ∗∗∗∗*p* < 0.0001. C-TMS, TMS treated with 5% (w/v) Chelex-100 resin; Fur, ferric iron uptake repressor.

Catecholamine hormones enhance growth of bacteria in serum by mediating iron release from transferrin (44, 46, 48, 54). The contribution of catecholamines to the growth of *S. aureus*, *via* SstABCD, is only evident when endogenous SA and SB biosynthesis is eliminated (55, 56). Given that *S. lugdunensis* does not produce a known siderophore, we hypothesized that the *sst* genes may contribute significantly to iron acquisition by this bacterium. Genome analysis of *S. lugdunensis* HKU09-01 revealed the presence of two tandem *sstABCD* loci that we designated *sst*-1 and *sst*-2 that share significant similarity at the nucleotide level but that are not identical. At the protein level, both Sst-1 and Sst-2 share significant identity with the SstABCD proteins in *S. aureus* suggesting both loci may play a role in catecholamine transport. Analysis of *sst*-1 and *sst*-2 promoter regions identified putative Fur boxes upstream of both *sstA* genes indicating iron-dependent regulation of gene expression from each *sst* locus (Fig. 3*A*). Indeed, qPCR analysis revealed that *sst-1* is highly upregulated in iron-deplete conditions as compared to iron-replete conditions consistent with Fur-dependent regulation of gene expression. In contrast, expression of the *sst*-2 locus was extremely low irrespective of the iron content in the growth medium suggesting the *sst-2* locus may be poorly expressed and therefore not function prominently in iron acquisition (Fig. 3*B*). To begin to characterize the importance of catecholamine-dependent iron acquisition and to determine the relative contribution of the *sst-1* and *sst-2* loci to *S. lugdunensis* growth, we created a *sst* deletion mutant lacking both *sst-1* and *sst-2* (Δ*sst*1/2); for unknown reasons, we could not successfully create single *sst*-locus deletion mutants. To confirm expression of Sst proteins in *S. lugdunensis*, we performed Western blot analysis on the bacteria cultured under iron replete and deplete conditions. Using antisera generated against the *S. aureus* SstD protein, we could immunodetect from WT *S. lugdunensis* a single protein at the expected SstD molecular weight, only under iron-deplete conditions (Fig. 3*C*) (44). That this anti-SstD reactive band is absent in the Δ*sst*1/2 mutant confirmed that this protein is indeed expressed from one of the Sst operons (Fig. 3*C*); likely SstD1 since gene expression from *sst*-2 was virtually undetectable.

To evaluate the ability of the Δ*sst*1/2 mutant to utilize catecholamines for growth under iron restriction, we compared the ability of WT *S. lugdunensis* and the Δ*sst*1/2 mutant to grow in iron-deplete medium when norepinephrine (NE) is provided as a sole source of iron (Fig. 3*D*). In the absence of NE, neither WT nor the Δ*sst*1/2 mutant could grow unless the culture medium was supplemented with FeCl_3_, which restored growth to both WT and mutant bacteria. In contrast, when the culture medium was supplemented with 50 μM NE, only WT *S. lugdunensis* was capable of growth indicating one or both *sst* loci enable NE utilization as an iron source (Fig. 3*D*).

**Figure 4:**
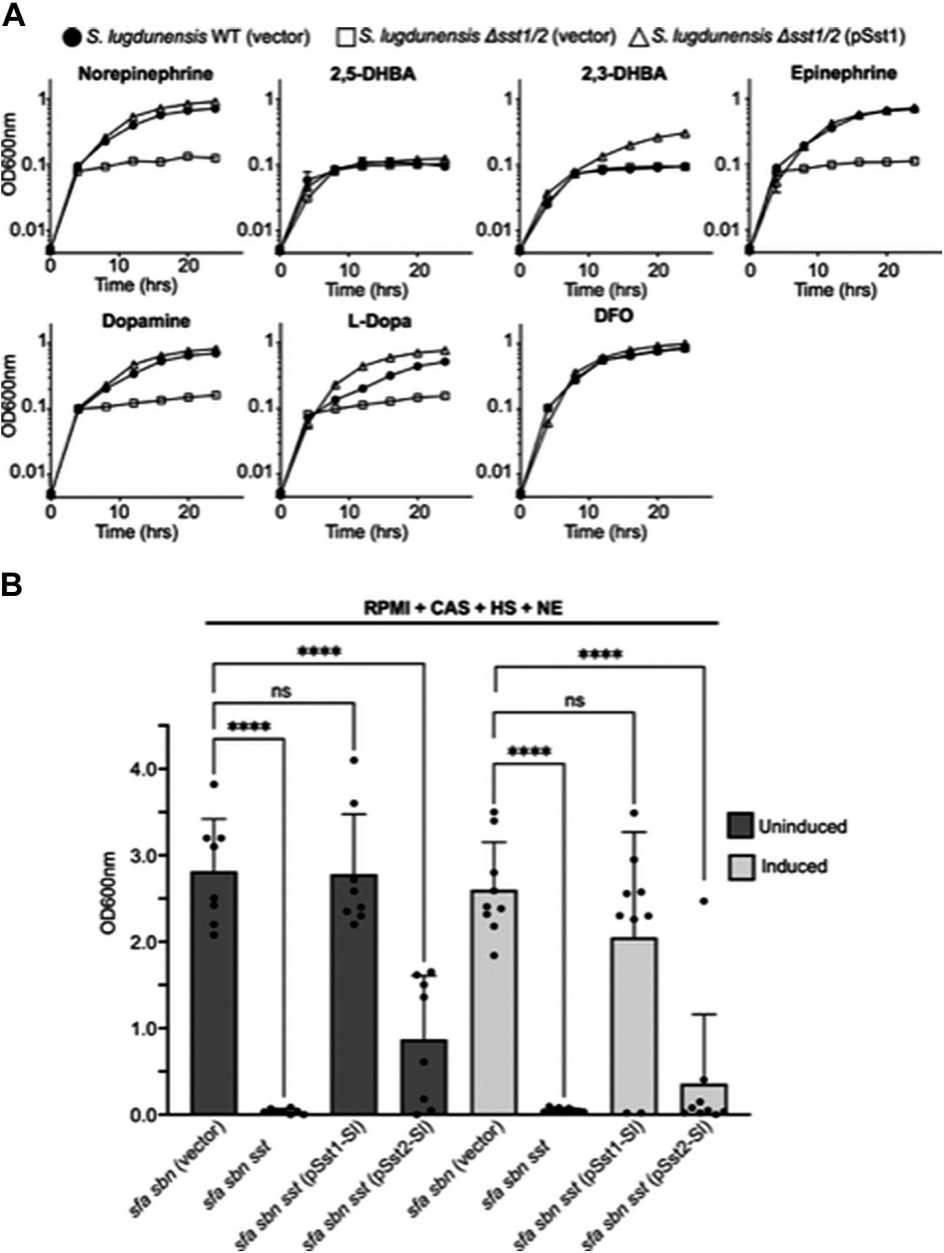
**The *sst*-1 locus of *S. lugdunensis* is required for use of host catecholamine stress hormones as iron sources.***A*, growth of *S. lugdunensis* deficient for the *sst*-1/2 loci (Δ*sst*-1/2) was assessed in the presence of human stress hormones. Growth was compared to WT *S. lugdunensis*, and Δ*sst*-1/2 carrying either a vector control or the pSst1 plasmid was analyzed in C-TMS with 20% (v/v) horse serum. Each catecholamine was supplemented at 50 μM each catechol compound indicated and desferrioxamine B (DFO), a hydroxamate-type siderophore, was used as a control. *B*, the ability of plasmids pSst1 or pSst2 (encoding the *sst* locus from *S. lugdunensis*) to promote growth of *sst*-deficient *S. aureus* strain Newman in the presence of 200 μM norepinephrine. Bacteria carrying the indicated mutations and plasmids were grown in RPMI supplemented with 1% (w/v) casamino acids and 0.2% (v/v) horse serum in the presence of antibiotic and aTc to induce *sstABCD* expression from either pSst1 or pSst2. The data shown are the mean ± standard deviation of three independent experiments where each symbol represents a biological replicate. Statistical significance was determined by ordinary one-way ANOVA with a Dunnett’s multiple comparison test using *sfa sbn*–deficient *S. aureus* as a comparator. In *B*, n.s. indicates not significant, ∗∗∗∗ *p* < 0.0001. C-TMS, TMS treated with 5% (w/v) Chelex-100 resin; DHBA, 2,3-dihydroxybenzoic acid; HS, horse serum; L-DOPA, L-3,4-dihydroxyphenylalanine.

We next wanted to determine the relative contributions of each *sst* operon to catecholamine utilization. Given we could not create single operon deletion mutants, we chose to complement the Δ*sst*-1/2 mutant with vectors carrying the individual *sst*-1 and *sst*-2 gene sets under the control of their native promoters. Provision of *sst*-1 operon in *trans* to the *Δsst*-1/2 mutant restored the ability of this strain to utilize NE and other catecholamines for growth akin to WT *S. lugdunensis* and as compared to the vector control (Fig. 4*A*). Surprisingly, although we could clone the *sst-2* locus in *Escherichia coli* and *S. aureus*, we repeatedly failed to successfully introduce this plasmid into *S. lugdunensis*. Indeed, the *S. lugdunensis* transformants that were recovered always contained plasmid carrying deletions within the cloned *sst*-2 region. Therefore, as an alternative means to determine whether the *sst*-2 locus contributes to catecholamine utilization, we analyzed the growth of three *S. lugdunensis* clinical isolates whereby genome analysis identified that these strains lack the *sst-1* locus while retaining the native *sst*-2 genes; indeed, of 20 clinical isolates analyzed by whole genome sequencing, three were found to lack the *sst-1* locus (see Table S2). As a control, we also included a clinical isolate where both *sst-1* and *sst-2* loci are intact. This analysis revealed that the *sst-1* positive isolate strain IVK84 grew in the presence of 50 μM NE. In contrast, all three isolates that are *sst-1* deficient failed to grow in the presence of NE despite encoding *sst-2* (Fig. S5*A*). That these strains fail to grow, due to iron insufficiency, is evident upon addition of 20 μM FeCl_3_ which restored growth to all three isolates. We also performed Western blot analysis on these clinical isolates to analyze expression of the SstD lipoprotein which underpins NE utilization. Interestingly, only bacterial cell lysate derived from clinal isolate IVK84 displayed an immune reactive band at the expected molecular weight (*i.e.*, ∼38 kDa) when probed with an anti-SstD antibody (Fig. S5*B*). In contrast, the three clinical isolates lacking *sst*1 but having an intact *sst*2 locus failed to react with SstD antisera demonstrating the SstD2 protein is not expressed in these bacteria (Fig. S5*B*).

In further pursuit of understanding the function of the *sst-2* locus encoded by *S. lugdunensis*, we also performed heterologous complementation experiments using *S. aureus* as this staphylococcal spp. could be transformed with, and maintain, the *sst-2* expression plasmid. To assess catecholamine utilization by *S. aureus*, we utilized a *sfa sbn sst* mutant that could not synthesize endogenous SA or SB, which can obscure catecholamine uptake as previously described (44). Plasmids carrying either the *sst*-1 or *sst*-2 operon (pSst1 and pSst2, respectively) from *S. lugdunensis* or the vector control were mobilized into *S. aureus sfa sbn sst* and assessed for growth under iron-restricted conditions in the presence of catecholamines. As a control growth of the *sfa sbn sst* mutant was compared to *S. aureus* lacking only the *sfa* and *sbn* genes (*i.e.*, SA and SB biosynthesis) and can be considered here as wildtype as it encodes an intact *sst* locus. In these experiments, *S. aureus* lacking *sfa sbn sst* but harboring pSst1 grew significantly better than the vector-control strain in medium supplemented with NE, epinephrine, dopamine, or L-3,4-dihydroxyphenylalanine (L-DOPA) (Figs. 4*B* and S6*A*). In contrast, the same *S. aureus sfa sbn sst* mutant harboring pSst2 was impaired for growth in presence of each catecholamine and grew akin to the vector control (Figs. 4*B* and S6*A*). As the *sst1* and *sst2* loci were cloned into the plasmid pRMC2, an anhydrotetracycline (aTc)-inducible plasmid, we also assessed complementation in the presence of aTc. Again, under these conditions (*i.e.*, with induction), the *sfa sbn sst* mutant of *S. aureus* carrying pSst2 failed to grow in the presence of NE (Fig. 4*B*). Interestingly, Western blot analysis of cell lysates from bacteria carrying pSst2 revealed this strain also fails to express SstD2 akin to the clinical isolates discussed above (Fig. S6*B*). Altogether, these findings indicate that the *sst*-2 locus in *S. lugdunensis* is not expressed under the conditions employed here explaining the observed inability of *S. lugdunensis* to utilize catecholamines for growth in an Sst2-dependent manner. In contrast, the *sst*-1 locus is both necessary and sufficient for catecholamine-iron acquisition in *S. lugdunensis*.

### SstD from *S. lugdunensis* binds catecholamines

**Figure 5:**
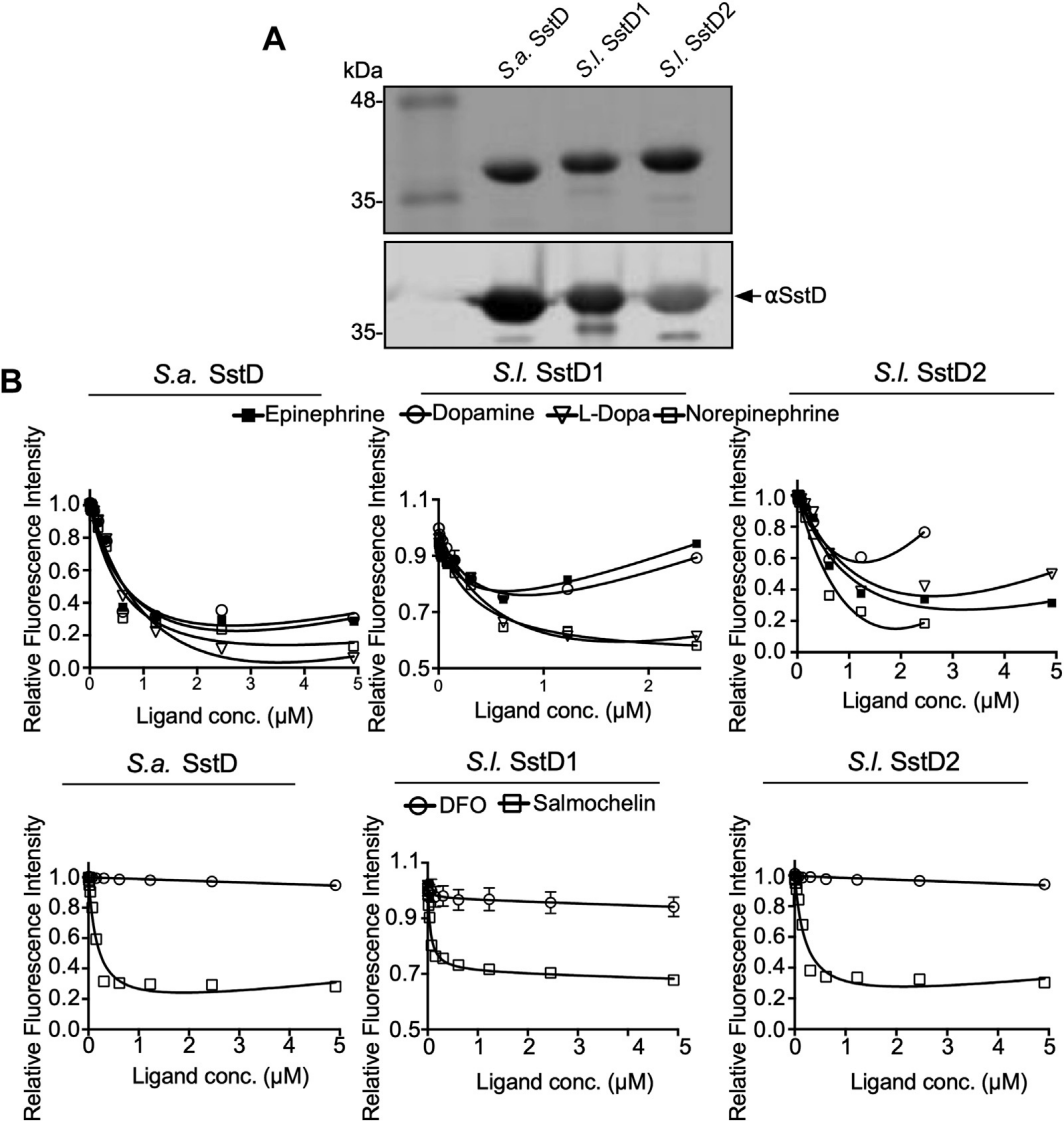
**The substrate-binding properties of *S. lugdunensis* SstD1 and SstD2 proteins.***A*, a Coomassie stains SDS-PAGE gel (*top panel*) shows the purity of the isolated recombinant SstD proteins from *S. lugdunensis* and *S. aureus*. Also shown is a representative Western blot (*bottom panel*) showing detection purified recombinant *S. aureus* (SA) and *S. lugdunensis* (SL) SstD homologs. Immunodetection was done with anti-SstD antiserum raised against *S. aureus* SstD protein. *B*, fluorescence quenching of recombinant SstD proteins in the presence of the indicated ferrated catecholamine hormones is shown. DFO and salmochelin were used as negative and positive controls respectively. Note: the inflection of the curves in the top panels is due to the intrinsic fluorescence of the catechol compounds that is evident at elevated concentrations of ligand, as has been previously observed (44). DFO, deferoxamine.

To biochemically characterize the *S. lugdunensis* SstD1 and SstD2, we investigated the substrate-binding of these two proteins. To this end, we overexpressed soluble forms of SstD1 and SstD2 from *S. lugdunensis* and SstD from *S. aureus* in *E. coli* and purified the proteins by metal-affinity chromatography. Antisera raised against *S. aureus* SstD recognized both *S. lugdunensis* SstD1 and SstD2 proteins in addition to the *S. aureus* protein (Fig. 5*A*). These proteins were then analyzed for substrate binding *via* intrinsic tryptophan fluorescence quenching. The fluorescence of all three SstD proteins was quenched in the presence of the catecholamines epinephrine, NE, dopamine, L-DOPA, and salmochelin but not in the presence of the hydroxamate DFO indicating specificity of SstD1 and SstD2 from *S. lugdunensis* and SstD from *S. aureus*, for binding catecholamines (Fig. 5*B*). Taken together, the ligand binding and biological growth data reveal that while both the SstD1 and SstD2 proteins of *S. lugdunensis* bind to catechol compounds, only the *sst1* locus contributes significantly to catecholamine-dependent iron acquisition under the culture conditions utilized herein.

### Sst-dependent utilization of an iron source in CAs

In characterizing iron acquisition by *S. lugdunensis*, experiments were performed where the bacteria were cultured in RPMI medium supplemented with 1% (w/v) CAs (RPMI-CAs). The addition of CAs is frequently used to promote growth of *S. aureus*, and we had previously attributed this phenomenon solely to the provision of additional amino acids. In our efforts to characterize catecholamine utilization by *S. lugdunensis*, we noted that the Δ*sst*-1/2 mutant would often fail to grow in RPMI-CAs. In contrast, WT *S. lugdunensis* grew optimally, and provision of pSst-1 restored growth to the Δ*sst*-1/2 strain indicating the observed defect was indeed *sst*-1 dependent (Fig. S7*A*). That supplementation of RPMI-CAs with FeCl_3_ also corrected the Δ*sst*-1/2 growth defect demonstrated this phenotype to be iron dependent. To determine whether this observation was specific to *S. lugdunensis*, we also performed similar experiments using established mutants of *S. aureus* that lacked either *sfa sbn*, *sst* alone, or *sfa sbn* and *sst* (44). This analysis revealed that *S. aureus* also utilizes a factor that is present in CAs for growth in a *sst*-dependent manner and that the apparent iron acquisition defect in *S. aureus* is only evident when endogenous siderophore production is perturbed (Fig. S7*B*). Given that the unknown factor present in CAs requires Sst catecholamine uptake systems in *S. lugdunensis* and *S. aureus* and that the growth defect is iron dependent, we speculated that commercially available CAs contains a catechol or related compound. To ascertain the identify of this factor, mass spectrometry was performed by two independent facilities to analyze the commercially available CAs. This analysis revealed that our stock CAs contained a compound or compounds related to the catecholamine methyl-DOPA, offering an explanation for the observed Sst-dependent growth in RPMI when supplemented with CAs.

### The ferrous iron transporter FeoAB allows *S. lugdunensis* to acquire iron at acidic pH

**Figure 6:**
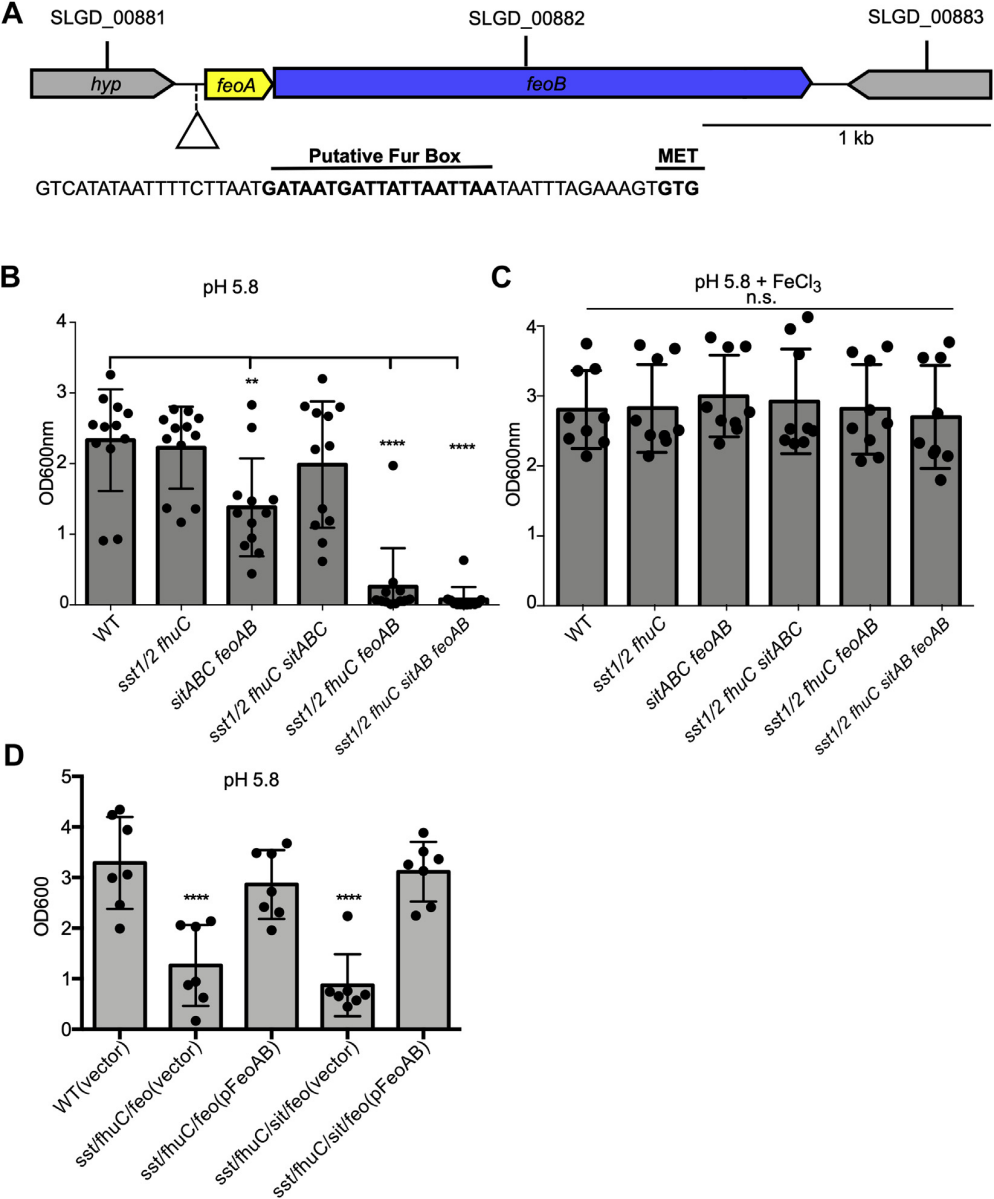
**The *S. lugdunensis* genes *feoAB* that encode a putative ferrous iron transporter are required for iron-dependent growth at acidic pH.***A*, the genetic organization of the *feoAB* locus in *S. lugdunensis* is shown. The nucleic acid sequence of the upstream promoter region is also shown, and the putative Fur box and alternative start codon to *feoA* is highlighted. *B* and *C*, growth of *S. lugdunensis* mutant lacking *feoAB* in the indicated genetic backgrounds is shown. The data are the mean ± standard deviation of the measured OD_600 nm_ after 24 h. In (*B*), the bacteria were grown in RPMI pH5.8, whereas in (*C*) the bacteria were grown in the same media but supplemented with 20 μM FeCl_3_. *D*, similar growth analysis was performed except the indicated *S. lugdunensis* strains were transformed with a vector control or the pFeoAB plasmid. *B*–*D*, each symbol represents a biological replicate, and the data derive from three independent experiments. Statistical significance was determined by a one-way ANOVA with a Dunnetts’s posttest where each dataset was compared to WT. n.s. indicates not significant, ∗∗*p* < 0.01, ∗∗∗∗*p* < 0.0001. Fur, ferric iron uptake repressor.

Analysis of the genome of *S. lugdunensis* HKU09-01 revealed the presence of the transport proteins that in other bacteria have been shown to play a role in the transport of divalent metal ions including ferrous (Fe^2+^) iron (Fig. 6*A*). Indeed, *S. lugdunensis* carries genes encoding the ferrous iron (Fe^2+^) transport system FeoAB (57, 58) as well as the metal ion transporter SitABC (MntABC) that, in *S. aureus*, has been shown to transport manganese (59). To characterize the contribution of *feoAB* and *sitABC* to *S. lugdunensis* growth, we created a series of deletion mutants where *feoAB* and/or *sitABC* were deleted from the bacterial genome. Growth analysis of these mutants was performed in RPMI-CAs acidified to pH 5.8 to maintain any trace iron in the ferrous (Fe^2+^) state which is suitable for FeoAB-dependent uptake. In this medium, *S. lugdunensis* lacking *sst*-1/2 and *fhuC* grew similarly to WT bacteria indicating that at pH 5.8 iron is more readily available (Fig. 6*B*). In contrast, a strain of *S. lugdunensis* lacking *feoAB* and *sitABC* demonstrated a modest yet statistically significant decrease in growth over a 24 h in the same culture medium. To ascertain whether *sitABC* or *feoAB* contributed significantly to iron acquisition at pH 5.8, these mutations were created in a Δ*fhuC* Δ*sst*-1/2 background. Growth of these mutants revealed that only when *feoAB* was inactivated, growth of *S. lugdunensis* lacking *fhuC* and *sst*-1/2 was ablated at pH5.8 (Fig. 6*B*). Moreover, the strains lacking *feoAB* failed to grow in an iron-dependent manner as supplementation of RPMI-CAs, pH 5.8 with FeCl_3_ restored growth of each strain to WT levels (Fig. 6*C*). To verify the observed phenotype was indeed dependent on *feoAB*, complementation was performed, and as expected, when *feoAB* was provided in *trans* under the control of the native promoter growth of *S. lugdunensis* was restored to WT levels at pH 5.8 (Fig. 6*D*).

### Comprehensive iron acquisition allows *S. lugdunensis* to proliferate in murine kidneys

**Figure 7:**
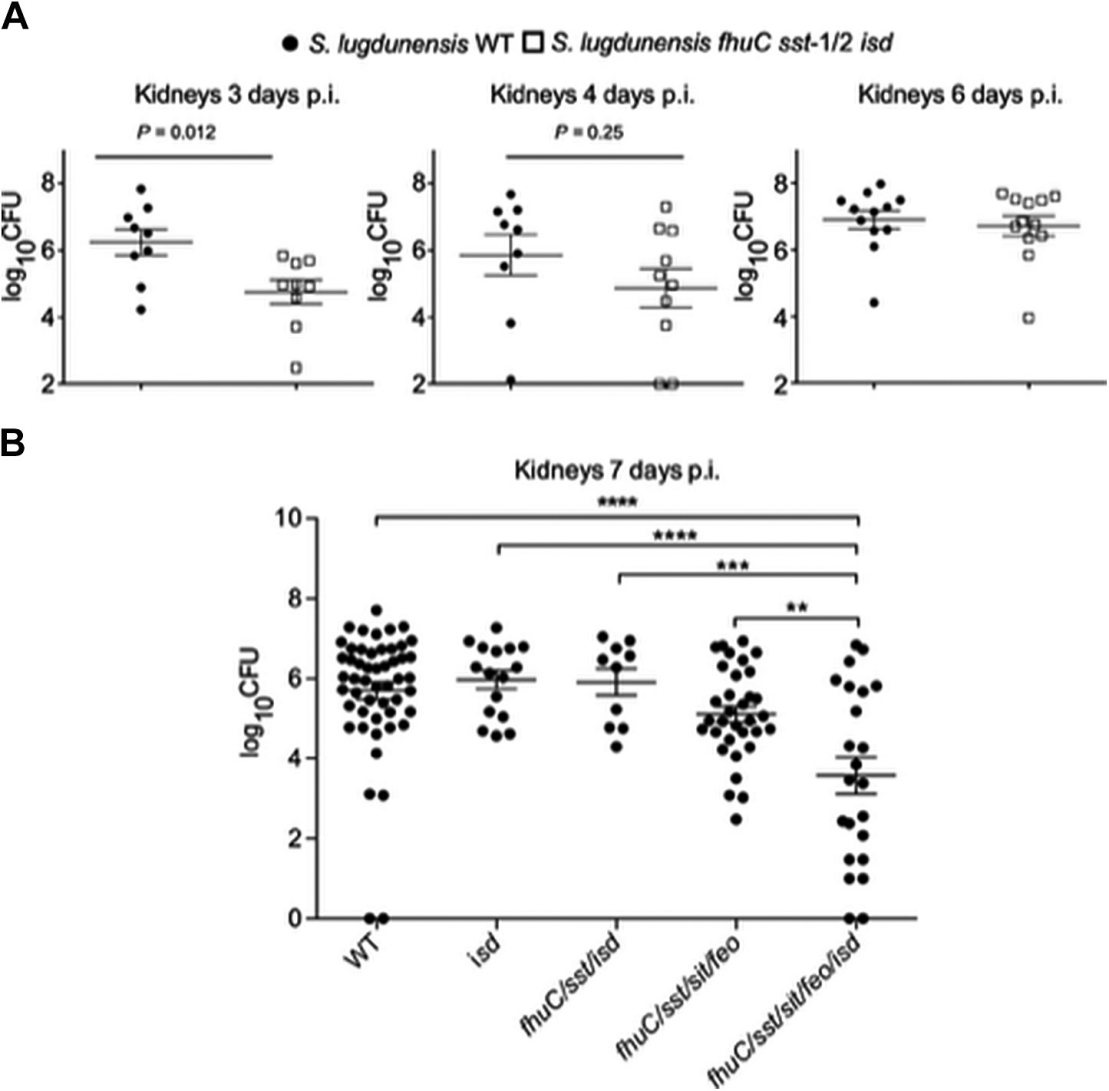
**Disruption of all of Isd, FhuC, Sst, Sit, and Feo in *S. lugdunensis* is required to attenuate bacterial growth in murine kidneys.** Female BALB/c mice were infected systemically with 2 to 3 × 10^7^ CFU *S. lugdunensis* WT or an isogenic *isd fhuC sst* mutant. *A*, the bacterial burden in the kidneys of infected mice is shown. Mice were sacrificed at day 3, 4, and 6, and each symbol represents the measured burden expressed as log10 CFU from both kidneys of a single mouse. The mean is depicted the horizontal bar and the error bars represent the standard error of the mean for each group. Statistical analyses were performed using an unpaired Student’s *t* test. The limit of detection is the y-axis value at the origin. *B*, the burden of *S. lugdunensis* strains lacking combinations of iron acquisition systems in the murine kidney at day 8 is shown. The data are presented as the mean log10 CFU/organ where the horizontal bar is the mean, and the bars are the standard deviation. Each *symbol* represents a single animal. Statistical significance was measured using an ordinary one-way ANOVA using a Tukey’s multiple comparison test. The *horizontal bars* indicate comparisons where statistically significant differences are observed and ∗∗*p* < 0.01, ∗∗∗*p* < 0.001, ∗∗∗∗*p* < 0.0001. CFU, colony-forming unit; Fhu, ferric hydroxamate uptake; Isd, iron-regulated surface determinant.

Iron is scarcely available within the mammalian host. The preceding experiments revealed the importance of several iron acquisition systems in *S. lugdunensis* and the conditions with which they function to acquire iron *in vitro*. However, we next sought to establish their importance during infection. Previous work from our laboratories has established that *S. lugdunensis* utilizes the Isd pathway as well as the LhaSTA transporter, encoded from within the *isd* locus, to acquire iron from heme and hemoglobin (35, 50). Given hemoglobin/heme are relevant sources of iron *in vivo*, we examined phenotypes for the *isd* mutant and, as shown in Figure 1, found that bacterial burdens in kidneys were no different than for that of mice infected with WT bacteria. Thus, other iron acquisitions mechanisms must be at play. Combining mutations in *sst* and *fhuC* into the *isd* mutant (the mutant had a confirmed inability to grow on hemin as a source of iron, Fig. S2) yielded a strain that was attenuated in the first several days of infection but eventually the bacterial burden reached levels similar to those seen in WT-infected mice (Fig. 7*A*). These data indicated that while *fhuC*, *sst*, and *isd* were involved in the early stages of infection in the kidneys, other iron acquisition systems must also function to allow the bacteria to eventually grow. Importantly however, these data indicate that *S. lugdunensis* must initially utilize catechol- and/or hydroxamate-type iron chelates that must exist within the host. To elucidate whether the *feo* and/or *sit* genes contribute to growth *in vivo*, we next incorporated mutations in these loci in the *fhuC sst isd* background and observed that this mutant was now attenuated (>2 log relative to WT) for proliferation in the kidneys through day 7 of the infection (Fig. 7*B*). In strains lacking mutations in either isd or *feo*/*sit*, there was no significant attenuation, indicating combined disruption of all of *isd*/*fhuC*/*sst*/*feo*/*sit* was required to establish long-lasting perturbation of infection in mice (Fig. 7*B*). Taken together, these data indicate that the *isd* genes in addition to the non-heme iron acquisition systems must operate in *S. lugdunensis* and work in concert to promote growth within murine kidneys.

## Discussion

**Figure 8:**
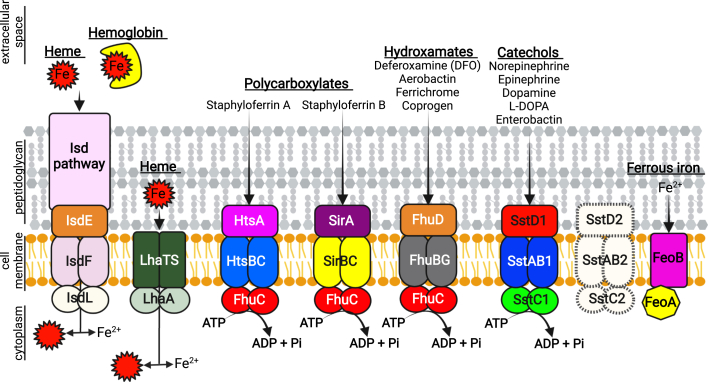
**The iron acquisition systems of *S. lugdunensis*.***S. lugdunensis* can utilize heme and non-heme sources of iron to support bacterial growth in iron-restricted environments including the mammalian host. The iron-regulated surface determinant (Isd) pathway that functions as a high-affinity heme iron acquisition system is depicted. The Isd pathway is comprised of peptidoglycan anchored proteins (represented by the *pink rectangle*) that can bind free heme or hemoglobin at the bacterial cell surface and extract heme. At the cytoplasmic membrane, heme is bound by the lipoprotein IsdE and transported to the interior of the cell through the permease IsdF. Iron (Fe) is liberated from heme by the heme oxygenase IsdG (not depicted). Heme can also be transported through the cytoplasmic membrane by an ECF-type transporter encoded by the *lhaSTA* genes. While *S. lugdunensis* does not synthesize siderophore, it can also utilize xenosiderophores (*i.e.*, siderophores made by other bacteria) to promote growth. The polycarboxylate siderophores staphyloferrin A and staphyloferrin B, made by *S. aureus*, can be taken up by the dedicated ABC-type transporters HtsABC and SirABC, respectively. Hydroxamate-type siderophores are transported through the FhuDBGC ABC-type transporter. The ATPase FhuC is utilized by Hts, Sir, and Fhu transport systems to hydrolyze ATP and provide the necessary energy for siderophore uptake. Catechol containing molecules such as norepinephrine, a host stress response hormone, are taken up by *S. lugdunensis* through the Sst1 ABC transporter encoded by the genes *sstABCD*1. The second Sst ABC-type transporter encoded by the *sstABCD2* genes (indicated by the *white shapes with dashed lines*) does not appear to be expressed in *S. lugdunensis* and does not support *S. lugdunensis* growth on catecholamines as a source of iron. *S. lugdunensis* can also utilize iron directly from the environment through the function of the ferrous iron (Fe^2+^) transporter FeoAB. Collectively, these iron acquisition systems enable *S. lugdunensis* to access a broad range of iron sources, thereby enabling the bacteria to acquire this essential trace metal. Figure created with BioRender.com. ECF, energy coupling factor; L-DOPA, L-3,4-dihydroxyphenylalanine.

The capacity to acquire iron underpins the ability of bacteria to cause infection, and *S. lugdunensis* thrives in diverse niches within the host where it can cause a spectrum of diseases. The ability of *S. lugdunensis* to cause disease necessitates this bacterium must deploy iron acquisition systems; however, unlike other staphylococci such as *S. aureus*, it does not produce a siderophore. Nevertheless, *S. lugdunensis* encodes within its genome, both heme- and non-heme–dependent iron acquisition systems that allow for proliferation when confronted with an intact mammalian immune system (summarized in Fig. 8). This is evidenced by the observation that the burden of *S. lugdunensis* within the murine kidney increases over time (see Fig. 1*C*) and that mutagenesis of iron acquisition genes can antagonize *S. lugdunensis* infection (see Fig. 7, *A* and *B*). Our infection data demonstrate that while inactivation of the Isd system alone in *S. lugdunensis* attenuates growth on hemin *in vitro*, Isd mutagenesis does not attenuate growth in the murine kidney. Ostensibly, this is because the IsdB protein of *S. lugdunensis* binds murine hemoglobin with reduced affinity as compared to human hemoglobin (34), and *S. lugdunensis* demonstrates weak hemolytic activity toward murine erythrocytes (36). Nonetheless, the underlying importance of the Isd pathway is evident when additional iron acquisition genes such as *fhuC*, *sst*-1/2, and *feoAB* are inactivated. Conversely, that an *isd* mutant alone also does not present with any defects *in vivo* as compared to WT indicates that these other non-heme iron acquisition systems must also operate *in vivo*. Collectively, the Fe acquisition pathways that function in *S. lugdunensis* must work in a concerted manner to provide sufficient iron to support *S. lugdunensis* growth in the murine kidney. Moreover, due to their overlapping function in metal acquisition, multiple mutations are required for *in vivo* phenotypes to be evident. Ostensibly, this is because different sources of iron exist within the kidney. Indeed, the kidney is a highly metabolic organ, and there is significant flux of iron that is ongoing. For instance, in the rat kidney, approximately 370 μg of iron is filtered daily and approximately 99.3% of iron filtered by the glomeruli are reabsorbed (60). Iron within the kidney can exist in the form of heme and heme-containing proteins (61, 62) and can be bound by the glycoprotein transferrin (63). Not surprisingly, the transport and metabolism of iron within the kidney is driven by a variety of host proteins such as the metal transporter Dmt1 (60, 64), heme metabolizing enzymes such as HO-1 (65, 66) and transferrin receptor 1–mediated endocytosis of iron-bound transferrin (63). Presumably, the concerted action of multiple *S. lugdunensis* iron acquisition systems ensures that the bacteria can access this critical metal within the host.

Based upon *in vitro* analyses, the importance of *S. lugdunensis* Fe acquisition using only specific iron sources has enabled us to demonstrate that this bacterium can transport a variety of siderophores, mammalian stress hormones, and even ferrous iron. That *S. lugdunensis* cannot synthesize a siderophore distinguishes this *Staphylococcus* spp. from *S. aureus* and other CoNS (35). Moreover, the inability of *S. lugdunensis* to synthesize siderophores explains why this bacterium is incapacitated for growth even in very low concentrations of HS (a source of transferrin) *in vitro* (see Fig. 1*A* and (35)). Despite not synthesizing siderophore, *S. lugdunensis* can usurp xenosiderophores (*i.e.*, siderophores produced by other bacteria). Indeed, *S. lugdunensis* can utilize hydroxamate-type siderophores such as aerobactin produced by *E. coli* and polycarboxylate siderophores such as SA and SB synthesized by *S. aureus* (see Fig. S3 and (35)). *S. lugdunensis* can also utilize catecholamine stress hormones that can interact with the host glycoprotein holotransferrin and liberate the bound iron rendering it available to the bacteria (49). The mechanism by which this occurs involves direct binding of catechols such as NE to holotransferrin and the subsequent reduction of transferrin-bound iron to the Fe^2+^ state which is poorly bound by the host glycoprotein (49). Catecholamines have been shown to promote growth of many other bacteria (49, 54) in addition to *S. aureus* under iron-limiting conditions. However, in the case of latter, the importance of catecholamine-dependent iron acquisition is only evident in the absence of endogenous staphyloferrin production (44). It is interesting that the *sfaA* and *sfaD* genes involved in SA biosynthesis and export are deleted in *S. lugdunensis* (35). Presumably, the inability to synthesize SA has unburdened *S. lugdunensis* with the need to consume metabolites and expend energy for siderophore biosynthesis, but why this would be of benefit to *S. lugdunensis* is unclear as the bacteria would be reliant on exogenous iron chelates.

*S. lugdunensis* strain HKU09-01 differs from *S. aureus* in that it encodes two tandem *sst* loci (*sst*-1 and *sst*-2) both of which were predicted to function in catecholamine use; however, our experiments clearly demonstrated that the ability of *S. lugdunensis* to derive iron from catecholamines can be attributed solely to the *sst*-1 locus. *S. lugdunensis* is unique among staphylococci in that it can carry duplicated *sst* gene sets, and this distribution of putative *sst* genes is seen in many clinical isolates (30, 31). It is interesting that three clinical isolates were identified that lack the *sst-1* locus; however, as our data reveal, these strains fail to utilize NE as an iron source, and it is unclear why such gene loss would occur in a background where siderophore is already not produced. Despite sst-1 clearly playing a role in catecholamine utilization, we fail to detect expression or show a function for *sst*-2 in catecholamine utilization or otherwise. That the SstD2 protein of *S. lugdunensis* can bind catecholamine substrates could suggest that the second *sst* locus in *S. lugdunensis* could in principle effect catecholamine utilization if SstD2 is expressed. At present, the mechanism that underlies our inability to detect expression of the *S. lugdunensis* SstD2 protein remains undefined. In contrast, our data clearly show that *sst*-1 allows this bacterium to utilize catecholamines, and perhaps in the absence of endogenous siderophore production, *S. lugdunensis* is often reliant on catechols for growth during infection as compared to *S. aureus*. It is interesting that *S. lugdunensis* displays growth in the murine kidney where catechols can indeed be produced and secreted from the renal tubules (67, 68). Conceivably, as *S. lugdunensis* lacks the ability to synthesize SA or SB, unlike *S. aureus*, this bacterium has evolved optimized SA- and SB-independent iron transport systems to compensate.

That *S. lugdunensis* cannot make siderophore might also render this bacterium more reliant on other siderophore-independent metal transporters such as FeoAB. Feo transporters function to transport ferrous iron into bacteria, and these systems have been better characterized in gram-negative organisms (58). In contrast, several gram-positive bacteria encode Fur-regulated Feo transport systems; however, their role in iron acquisition has remained largely uncharacterized (22). Here, we show for the first time for a *Staphylococcus* spp. that FeoAB is required for growth at acidic pH in an iron-dependent manner. Under these conditions (*i.e.*, pH 5.8), iron should exist in the Fe^2+^ state, and *in vivo* Fe^2+^ might exist in hypoxic/anoxic or acidic environments such as abscesses or phagolysosomes within immune cells. In contrast to FeoAB, a role for the *sitABC/mntABC* locus in iron acquisition in *S. lugdunensis* could not be found *in vitro* indicating FeoAB is primarily utilized by this organism for Fe^2+^ utilization.

During our investigation, we found that *S. lugdunensis* displayed improved growth in the presence of CAs used to supplement RPMI. Naively, we initially attributed the improved growth of *S. lugdunensis* to the provision of additional amino acids; however, we found that improved growth of *S. lugdunensis* in RPMI with CAs was dependent on the *sst*-1 locus. Moreover, compromised growth of a *sst* mutant could be rescued by the addition of iron not amino acids. These observations indicated that CAs provided the bacteria with an additional iron source and mass spectrophotometry confirmed that catecholamines, such as methyl-DOPA, can be present in commercial CAs preparations. As CAs are often used to supplement bacterial media such as Tris-minimal succinate or RPMI (44, 69, 70), caution should be taken as impurities present in CAs could have unintended effects on experimental outcome.

*S. lugdunensis* lacks the vast arsenal of virulence factors present in *S. aureus*, which may explain why the bacterial burden in the liver of infected animals decreases with time. In contrast, the burden of *S. lugdunensis* does increase in the kidney over time (see Fig. 1*C*). Previously, it has been shown that a heme auxotroph of *S. aureus* is better able to replicate within the murine kidney, as compared to other visceral organs, suggesting the kidney might be a niche where heme is more readily availability (51). Therefore, *S. lugdunensis* might be poised to grow in this organ as it expresses both Isd and an ECF-type ABC transporter specific for heme (35, 36, 50). Both systems are Fur-regulated and therefore expressed under iron-limiting conditions (*i.e.*, *in vivo*); however, other low affinity heme transport systems might also exist. Interestingly, the Isd proteins and ECF heme transporter in *S. lugdunensis* enable growth on heme of murine and human origin; however, the hemolytic factors secreted by *S. lugdunensis* have a propensity to lyse only human erythrocytes (36). Indeed, studies have reported that in a systemic murine model of infection, *S. lugdunensis* fails to cause significant morbidity (20, 21), an observation corroborated here, which could be in part be attributable to the presence of human specific hemolysins. Nevertheless, animal models are an important tool that can be employed to study *S. lugdunensis* growth and metal acquisition in the mammalian host and that *S. lugdunensis* failed to cause significant morbidity facilitated our study. Indeed, through *in vivo* infection experiments, we have demonstrated that several genetic loci within *S. lugdunensis* allow the bacteria to procure iron from an assortment of non-heme–related and heme-related sources *in vitro*; however, *in vivo*, it is the concerted action of these systems that allows *S. lugdunensis* to grow. Therefore, the development of interventions that target bacterial iron acquisition systems should consider the overlapping function of distinct metal acquisition strategies deployed by bacterial pathogens.

## Experimental procedures

### Bacterial strains and media

Bacterial strains and vectors employed in this study are summarized in Table S1. *E. coli* strains were grown in Luria-Bertani broth (LB, BD Diagnostics) or on LB agar. For routine culture and genetic manipulation, *S. lugdunensis* and *S. aureus* strains were cultured in tryptic soy broth (TSB) or on TSB solidified with 1.5% (w/v) agar (TSA). For growth experiments, *S. lugdunensis* and *S. aureus* were cultured in RPMI 1640 (Life Technologies) that was in some instances supplemented with 1% (w/v) CAs (BD Diagnostics) (RPMI-CAs). Growth was also performed in Tris-minimal succinate (TMS) broth or 1.5% (w/v) agar (71). TMS broth was treated with 5% (w/v) Chelex-100 resin (Bio-Rad) at 4 °C for 24 h (C-TMS) to chelate trace metals. As appropriate, the above media were supplemented with heat-inactivated HS (Sigma Aldrich) which served as a source of transferrin to restrict iron availability. Bacteria were cultured at 37 °C with shaking at 220 rpm unless otherwise indicated. For *E. coli*, antibiotic selection was as follows: 100 μg ml^−1^ ampicillin or 50 μg ml^−1^ kanamycin. For *S. lugdunensis* and *S. aureus*, antibiotic selection was as follows: 10 to 12 μg ml^−1^ chloramphenicol, 4 μg ml^−1^ tetracycline, and 50 μg ml^−1^ kanamycin. aTc to induce expression from pRMC2 plasmids was used at 250 ng/ml.

### Real-time PCR

Quantitative real-time PCR was performed as previously described (72). Briefly, *S. lugdunensis* HKU09-01 RNA was prepared from triplicate 3 ml cultures grown in C-TMS or C-TMS with 100 μM FeCl_3_. Cultures were harvested to an OD_600_ of 3.0, and RNA was extracted using the Aurum Total RNA Mini Kit (BioRad). Extracted RNA (500 ng) was reverse-transcribed and PCR-amplified using iScript One-Step RT-PCR Kit with SYBR Green (Bio-Rad) and primers outlined in Table S1. Data were normalized relative to expression of the *rpoB* housekeeping gene.

### Gene deletion and complementation of *S. lugdunensis*

For in-frame deletion of *S. lugdunensis* genes, allelic replacement using the pKOR1 or pIMAY vector was performed as described previously (73, 74). Briefly, 500- to 1000-bp DNA fragments flanking regions of interest were amplified using the primers found in Table S1. Upstream and downstream flanking amplicons were cloned into pKOR1 or pIMAY. Knockout vectors were passed through *S. aureus* RN4220 or *E. coli* SL01B before introduction into *S. lugdunensis* by electroporation (21, 74). Plasmids were integrated into the genome at 42 °C for pKOR1 or at 37 °C for pIMAY in the presence of chloramphenicol prior to counter-selection at 30 °C in the presence of aTc (pKOR1: 200 ng ml^−1^, pIMAY: 1 μg ml^−1^). Chloramphenicol-sensitive colonies were chosen for screening by PCR across the deleted region in the chromosome, which was further confirmed by sequencing (73, 74). The same process was used over to generate multiple deletions in one strain.

For complementation, the *S. lugdunensis fhuC*, *sstA1B1C1D1* and *sstA2B2C2D2*, and feoAB genes were PCR amplified using primers described in Table S1, and each amplicon encompassed the native upstream promoter. The *fhuC*, *sstA1B1C1D1*, and *sstA2B2C2D2* amplicons were cloned into the plasmid pRMC2 to create of pFhuC, pSst1, and pSst2. The *feoAB* amplicon was cloned into the plasmid pALC2073. Each amplicon was cloned as a KpnI/SacI fragment, and plasmids were confirmed by DNA sequencing. All cloning was performed in *E. coli* DH5α, and the resulting plasmids were passed through *S. aureus* RN4220 or *E. coli* SLO1 prior to electroporation into the appropriate *S. lugdunensis* mutant.

### Whole genome sequencing

*S. lugdunensis* genomic DNA was isolated using the PurElute Bacterial Genomic Kit (Edge Biosystems) with an additional incubation step with lysostaphin (25 μg/ml) at 37 °C for 10 min. Libraries were created using the Nextera tagmentation kit (Illumina) and sequenced on an Illumina MiSeq with paired-end sequencing (2 × 241 bp). DNA sequence reads were assembled using nf-core pipeline bacass (v2.0.0 (75, 76)). Assembled scaffolds were annotated using PGAP (NCBI, v.2021-11-29.build5742 (77, 78, 79)) to obtain highly accurate annotations and Prokka (v1.14.6 (80)) to annotate genes similar to *S. lugdunensis* N920143 (RefSeq assembly accession: GCF_000270465.1). To have N920143 gene names in the Prokka gff output file, the --*proteins* option was used with a modified gbk file, where the CDS/gene attribute was replaced by the CDS/locus_tag attribute. N920143 annotations from prokka were merged with PGAP annotations using R (v4.1.2 (81) with packages dplyr, tidyverse, and fuzzyjoin). Gene positions were matched, allowing for a difference of up to three amino acids at either the start or end position. This loose match filter allowed us to account for slight differences in predicted start positions and annotate an additional 2.5% of genes with N920143 annotations. N920143 annotation was added as a CDS\note attribute in the PGAP annotation. Finally, annotations were curated using NCBI Genome Workbench (v3.7.0 (82)).

### Siderophore preparation and plate bioassays

*S. aureus*-concentrated culture supernatants were prepared from Δ*sbn*, Δ*sfa*, and Δ*sbn*Δ*sfa* mutants, respectively, as described previously (38). Strains were grown in C-TMS with aeration for 36 h before removal of cells. Supernatants were lyophilized, and insoluble matter was removed by methanol extraction (one-fifth original culture volume). Methanol was removed by rotary evaporation, and dried material was resuspended in water to one-tenth culture volume to provide culture extracts. SB was prepared *in vitro* enzymatically, as described previously (39, 42, 83). Enzymes were removed from the reaction mixture using an Amicon Ultra-0.5 10k filter column (Millipore), and the SB reaction mixture was normalized to DFO (London Health Sciences Center) equivalents as determined using the chrome azurol S (CAS) siderophore detection assay (84). SA was commercially prepared by Indus BioSciences (India). Ferric–enterobactin, –salmochelin S4, –aerobactin, and coprogen were purchased from EMC Microcollections. Ferrichrome was purchased from Sigma, whereas citrate was purchased from Fisher Scientific.

The ability of culture supernatants and purified siderophores to support *S. lugdunensis* iron-restricted growth was assessed with agar plates using plate-based disk diffusion bioassays (35, 43). Briefly, 1 × 10^4^ *S. lugdunensis* cells were incorporated into TMS agar containing 5 μM ethylenediamine-di(o-hydroxyphenylacetic acid) (EDDHA, LGC Standards GmbH). Siderophores/supernatants applied to sterile paper disks were placed onto the agar, and growth around disks was measured after 24 h at 37 °C.

### SstD Western blot analysis

Antisera against *S. aureus* SstD, used in this study, was previously prepared (44) and was used for analysis SstD expression in *S. lugdunensis*. *S. lugdunensis* bacteria were grown in C-TMS with or without 100 μM FeCl_3_ for 24 h, normalized, and lysed in the presence of lysostaphin (Sigma). Whole cell lysates were normalized to 8 μg total protein and resolved by SDS-polyacrylamide gel electrophoresis. For the detection of SstD expression in clinical isolates of *S. lugdunensis* or in pSst1/pSst2 carrying *S. aureus*, the bacteria were grown in RPMI-CAs and in the presence of chloramphenicol and aTc (250 ng/ml) as appropriate. Bacterial cell lysates were prepared as previously described (51). Western blotting was performed as previously described (35). In brief, the membrane was blocked in phosphate buffered saline (PBS) with 10% (w/v) skim milk and 0.05% (v/v) Tween 20. Antiserum was applied at a 1:500 dilution in PBS with 0.05% Tween 20 and 0.5% skim milk and incubated with nitrocellulose membrane overnight at in the cold. Anti-rabbit IgG conjugated to IRDye-800 (1:10,000 dilution; Li-Cor Biosciences). Fluorescence imaging was performed using a Li-Cor Odyssey infrared imager (Li-Cor Biosciences), and the resulting Western blots were contrast enhanced using Image J.

### Growth in serum

Growth of *S. lugdunensis* and *S. aureus* strains was assessed in C-TMS with serum. Single, isolated colonies were resuspended in 2 ml C-TMS and grown for over 4 h until OD_600_ was above 1. Each culture was normalized to an OD_600_ of 1 and subcultured 1:200 in C-TMS:HS. WT *S. lugdunensis* as well as *S. aureus* strains bearing Δsbn and Δsfa mutations are impaired for growth in this media compared to siderophore-producing strains (35, 38). Human stress hormones were added to the media for a final concentration of 50 μM to assess for catecholamine–iron acquisition for growth enhancement. Dopamine hydrochloride, L-DOPA, DL-NE hydrochloride, (-)-epinephrine, 2,3-dihydroxybenzoic acid (DHBA), and 2,5-DHBA were purchased from Sigma. Chloramphenicol was also included for strains harboring pRMC2 or derivatives. Cultures were grown in a Bioscreen C plate reader (Growth Curves USA) at 37 °C with constant shaking at medium amplitude. OD_600_ was assessed at 15-min intervals; however, for graphical clarity, 4 h intervals are shown.

### Protein overexpression and purification

Recombinant *S. aureus* SstD was purified as previously described (44). Regions of the genes encoding the soluble portions of *S. lugdunensis* SstD1 and SstD2 (*i.e.*, without the signal sequence and lipobox motifs) were amplified and cloned into pET28(a)+ (Novagen) using primers listed in Table S1. *E. coli* BL21 bearing pET28::*sstD1* or pET28::*sstD2* were grown to mid-log phase at 37 °C in LB with kanamycin, prior to induction with 0.4 mM isopropyl-β-D-1-thiogalactopyranoside (IPTG). After addition of IPTG, cultures were grown at 25 °C overnight. Cells were collected by centrifugation, resuspended in 20 mM Tris, pH 8.0, 500 mM NaCl, 10 mM imidazole (binding buffer), and ruptured in a cell disruptor (Constant Systems Ltd). Insoluble matter and debris were removed by centrifugation at 3000*g* for 15 min, followed by 150,000*g* for 60 min, sonicating samples in between. Soluble material was filtered and applied to a nickel-loaded 1 ml HisTrap column (GE Healthcare) equilibrated with binding buffer. His_6_-tagged proteins were eluted in 1 ml fractions from the column over a 0 to 80% gradient of 20 mM Tris, pH 8.0, 500 mM NaCl, 500 mM imidazole (elution buffer). Fractions bearing pure SstD1 and SstD2 (analyzed *via* SDS-PAGE) were pooled and dialyzed into 10 mM Tris, pH 8.0, 100 mM NaCl (working buffer) at 4 °C. Protein concentrations (Bio-Rad protein assay) were normalized to equality, and aliquots were frozen at −80 °C.

### Protein–ligand binding

Intrinsic tryptophan fluorescence quenching was used to assess protein–ligand binding affinity for *S. lugdunensis* SstD1, SstD2, and *S. aureus* SstD as previously described (44). Proteins were adjusted to 0.5 μM in 3 ml working buffer, and ligands were added at 2-fold concentration increments. Dopamine, L-DOPA, epinephrine, NE, DFO, and salmochelin S4 were used as ligands. Ligands were incubated in 3:1 (catecholamine hormones) or 1:1 (siderophores) molar ratio to FeCl_3_ for 5 min at room temperature prior to use. Bovine serum albumin (Sigma) was used as a protein negative control. Fluorescence was measured at room temperature in a Fluorolog instrument (Horiba Group), with excitation at 280 nm and emission detection at 345 nm. An excitation slit width of 5 nm and an emission slit width of 5 nm were used. Changes in fluorescence due to ligand additions and sample volume increase were corrected for (85). Fluorescence intensity data analysis was performed as previously described (44).

### Analysis of recombinant protein expression

Recombinant proteins were analyzed for purity and immunogenicity toward αSstD (*S. aureus*) antisera. *S. lugdunensis* SstD1 and SstD2 and *S. aureus* SstD purified protein volumes were normalized to contain 3 μg total protein and resolved by SDS-PAGE. Western blotting was performed as described above with the following modifications. After blocking, αSstD antisera were applied at a 1:10,000 dilution, and αHis antibody was applied 1:10,000. Anti-rabbit IgG conjugated to IRDye-800 (1:20,000 dilution) was secondary to αSstD antisera, whereas anti-mouse Alexa Fluor 680 (Life Technologies) was secondary to αHis (1:20,000 dilution). Antibodies/antisera were applied in PBS with 0.05% Tween 20 and 5% HS.

### Analysis of *S. lugdunensis* growth under iron restriction

*S. lugdunensis* with or without plasmids were grown O/N at 37 °C on TSA plates in the presence of selection as appropriate. Isolated colonies were resuspended in 2 ml of growth medium (*i.e.*, C-TMS or RPMI) in 14 ml polypropylene snap cap tubes and grown overnight at 37 °C with shaking at 225 rpm. Each culture was pelleted and washed twice in sterile 0.9% (w/v) saline and normalized to an OD_600_ of 0.5. Next day, 2 ml cultures were set up in 14 ml polypropylene snap cap tubes containing RPMI, RPMI-C, or RPMI at pH5.8 (acidified with HCl) with or without HS as necessary. Cultures were inoculated at a starting OD_600_ of 0.005 and were grown for 18 to 24 h at 37 °C with shaking at 225 rpm. Endpoint OD_600_ was read to evaluate the ability of *S. lugdunensis* to grow. In some instances, the additive DFO (100 μM), NE (50 μM or 100 μM), or hemin (50 nM) or FeCl_3_ (20 μM) was added to some cultures.

Growth curves were monitored using a BioScreen C plate reader with constant shaking at medium amplitude at 37 °C. OD_600_ was measured at 15-min intervals, and growth at 4-h intervals are shown. Here, growth of *S. lugdunensis* and *S. aureus* strains was assessed in C-TMS with serum. Isolated colonies were resuspended in 2 ml C-TMS and grown for over 4 h until OD_600_ was above 1. Each culture was normalized to an OD_600_ of 1 and subcultured 1:200 in C-TMS:HS. Cultures were pipetted into BioScreen C honeycomb plates in 200 μl culture volumes. WT *S. lugdunensis* as well as *S. aureus* strains bearing Δsbn and Δsfa mutations are impaired for growth in this media compared to siderophore-producing strains (35, 38). Human stress hormones were added to the media for a final concentration of 50 μM to assess for catecholamine–iron acquisition for growth enhancement. Dopamine hydrochloride, L-DOPA, DL-norepinephrine hydrochloride, (-)-epinephrine, DHBA, and 2,5-DHBA were purchased from Sigma.

### Murine model of systemic *S. lugdunensis* infection

All protocols for murine infection were reviewed and approved by the University of Western Ontario’s Animal Use Subcommittee, a subcommittee of the University Council on Animal Care. Six-week-old, female, BALB/c mice were obtained from Charles River Laboratories and housed in microisolator cages. *S. lugdunensis* strains were grown to mid-exponential phase (OD_600_ 2–2.5) in 25 ml TSB, washed twice with PBS, and resuspended in PBS to an OD_600_ of 0.50. Next, 100 μl of bacterial suspension, equivalent to ∼2 to 3 × 10^7^ colony-forming unit (CFU), was injected into each mouse *via* tail vein. Mice were weighed at time of challenge and every 24 h after, where infection was allowed to proceed for up to 8 days as necessary before mice were euthanized *via* cervical dislocation. Organs were aseptically harvested into 3 ml PBS with 0.1% (v/v) Triton X-100, homogenized, diluted, and plated onto TSA to enumerate bacterial burden. Weight data are presented as the difference in percentage from mouse weight at time of challenge. Recovered bacterial load from organs is presented as log_10_ CFU per organ.

### Human hemoglobin purification

Human hemoglobin was purified as described elsewhere (86).

### Analysis of *S. lugdunensis* growth with human hemoglobin

*S. lugdunensis* N920143 WT, Δ*isdL*, Δ*fhuC*, and Δ*isdL*Δ*fhuC* was grown O/N in TSB at 37 °C with shaking at 160 rpm. Cells were pelleted, washed with RPMI-CAs and 10 μM EDDHA, and adjusted to OD_600_ equal to 1. 2.5 μl of these cultures were used to inoculate 500 μl of RPMI +1% casamino acids +10 μM EDDHA per well (starting OD_600_ of 0.005) in a 48-well microtiter plate (Nunc, Thermo Scientific). As iron sources, 2.5 μg/ml human hemoglobin (hHb, own preparation) or 20 μM FeSO_4_ (Sigma-Aldrich) were added. Growth was measured using an Epoch2 reader (BioTek) (37 °C, orbital shaking) every 15 min for 48 h.

### Plasmid constructions for bacterial adenylate cyclase two-hybrid system

*S. lugdunensis* N920143 WT chromosomal DNA was used to amplify *isdF*, *isdL*, and *fhuC*, and the fragments were cloned into the vectors pKT25 and pUT18C (Euromedex), respectively, by restriction digestion. Used primers can be found in Table S1. After transformation into *E. coli* XL-1 blue, colonies were confirmed by sequencing.

### Bacterial adenylate cyclase two-hybrid system assay

To investigate interaction between the permease IsdF and the ATPases IsdL and FhuC, the commercially available bacterial adenylate cyclase two-hybrid system kit was used (Euromedex). In brief, *E. coli* BTH101 was co-transformed with pKT25:*isdF* and pUT18C:*isdL* or pUT18C:*fhuC*, respectively. In case of protein–protein interaction, the catalytic domains T25 and T18 of the *Bordetella pertussis* adenylate cyclase are able to heterodimerize and to produce cyclic AMP allowing the expression of *lacZ*. This leads to blue colony formation on LB agar indicator plates containing 40 μg/ml X-Gal (Sigma-Aldrich/Merck), 0.5 mM IPTG (Thermo Scientific), 100 μg/ml ampicillin, and 50 μg/ml kanamycin after incubation for 2 days at 30 °C. As positive control, pKT25:*zip* and pUT18C:*zip* were used encoding a leucine zipper; as negative control, empty vectors were co-transformed into BTH101.

## Data availability

Annotated *S. lugdunensis* clinical isolate genome sequencing data were deposited in the NCBI BioProject under accession PRJNA796272.

## Supporting information

This article contains supporting information (21, 30, 31, 35, 44, 73, 74, 87, 88, 89).

## Conflict of interest

The authors declare that they have no conflicts of interest with the contents of this article.
