# Supplementary material for: In vivo growth of Staphylococcus lugdunensis is facilitated by the concerted function of heme and non-heme iron acquisition mechanisms
Source: J Biol Chem. 2022 Mar 10;298(5):101823. doi: 10.1016/j.jbc.2022.101823 (PMC9052147; doi:10.1016/j.jbc.2022.101823)
Supplement: Supplemental Figures S1–S7 and Table S1 [file mmc1.docx]

**Supporting information**

**In vivo growth of *Staphylococcus lugdunensis* is facilitated by the concerted function of heme and non-heme iron acquisition mechanisms**

Ronald S. Flannagan, Jeremy R. Brozyna, Brijesh Kumar, Lea A. Adolf, Jeffrey John Power, Simon Heilbronner and David E. Heinrichs

Supporting information files include:

Figure S1

Figure S2

Figure S3

Figure S4

Figure S5

Figure S6

Figure S7

Table S1

Table S2

**
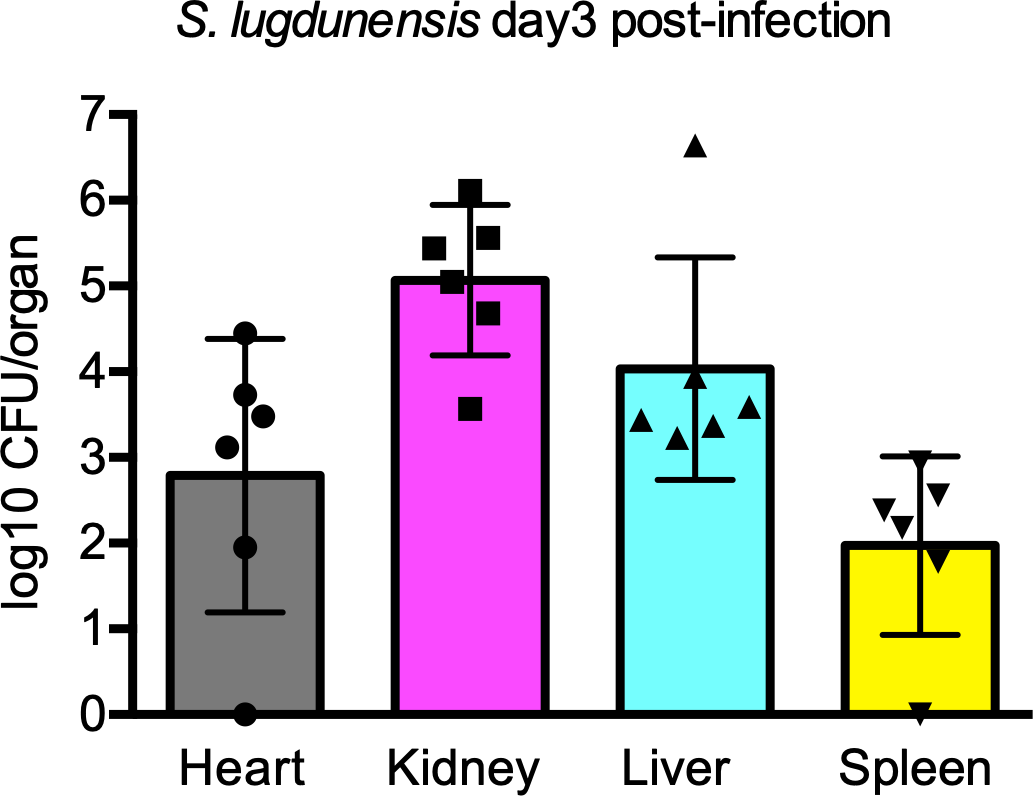
**

**Figure S1. Analysis of *S. lugdunensis* burden in visceral organs during systemic murine infection.** Mice were infected with 3.8 x 10^7^ of wild-type *S. lugdunensis* and the bacterial burden in the indicated organs was determined 3 days (72 h) post-infection where each symbol represents an individual animal that was infected. The data presented are the mean ± standard deviation for the determined log 10 CFU/organ.

**
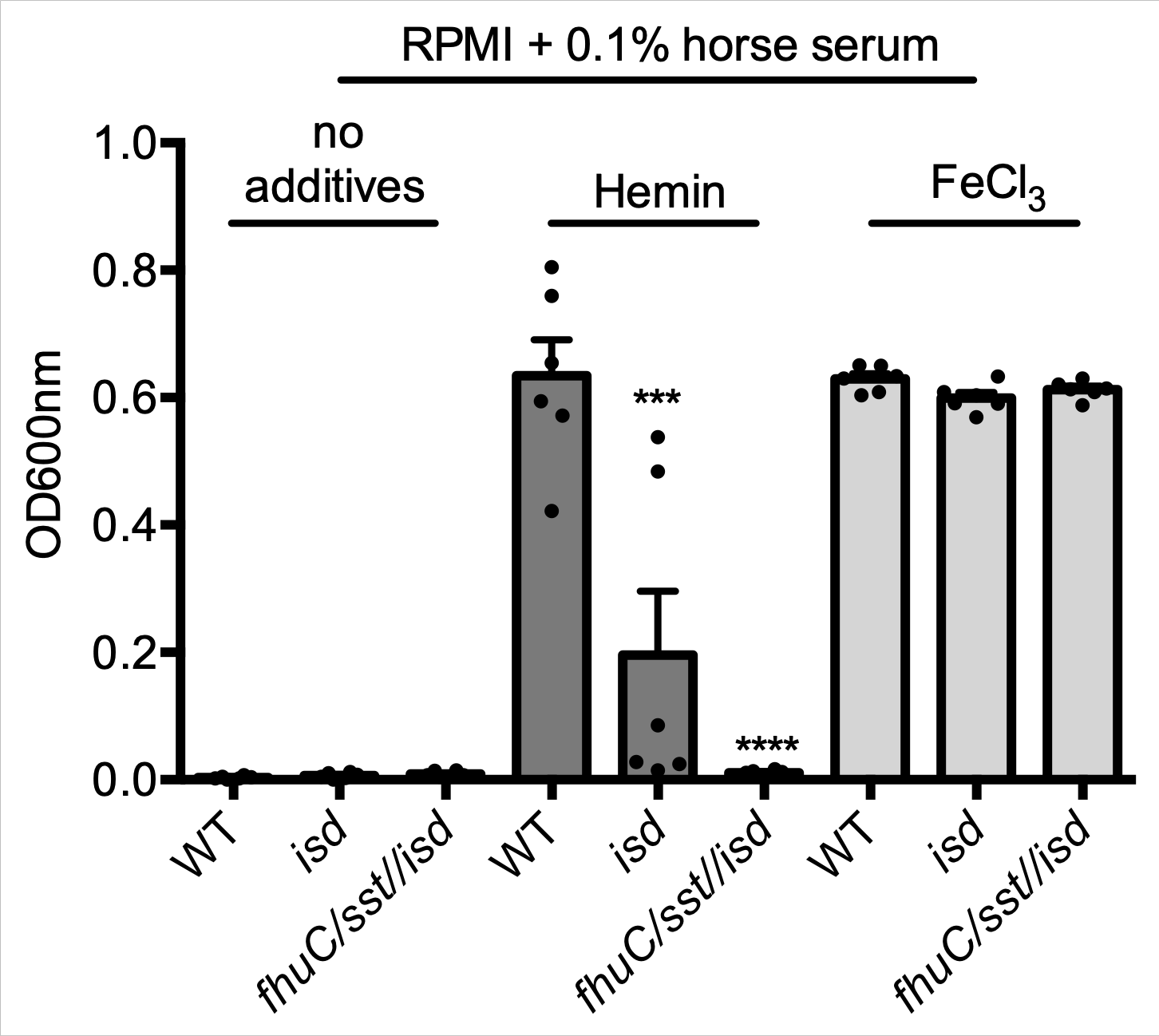
**

**Figure S2. *S. lugdunensis* lacking the *isd* locus cannot utilize hemin as an iron source.** Wild-type *S. lugdunensis* HKU09-01 and mutants lacking *isd* were cultured in RPMI supplemented with 0.1% (v/v) horse serum. The bacteria were also grown in the presence of 50 nM hemin or 20 μM FeCl_3_. The data presented are the mean ± standard deviation of the endpoint OD600nm measured at 24 h. Each data point represents a separate biological replicate from three independent experiments. Statistical significance was determined using an ordinary one-way ANOVA with a tukey’s multiple comparison test. ***p<0.001, ****p<0.0001.

**
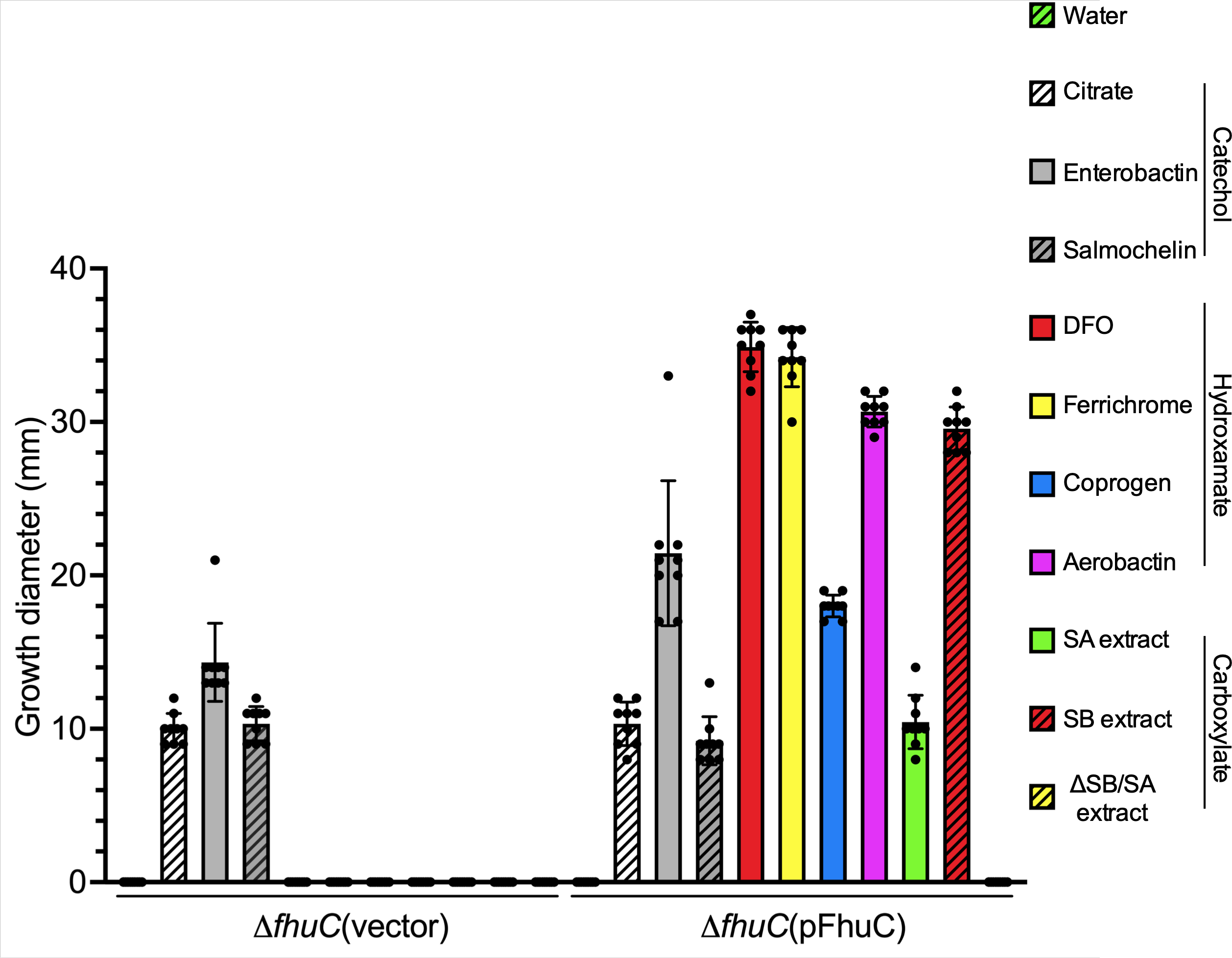
**

**Figure S3.** **The *fhuC* gene of *S. lugdunensis* is required for hydroxamate and staphyloferrin siderophore utilization.** Show is the mean diameter of growth for the *fhuC* mutant carrying either vector control or the pFhuC plasmid. Growth was measured around sterile paper discs that were impregnated with the indicated iron source or sterile Milli-Q water as a negative control. The data from the plate bioassays demonstrate that a *S. lugdunensis* *fhuC* mutant can use a catecholamine siderophores but cannot utilize hydroxamate-bound or staphyloferrin-bound iron. The hydroxycarboxylates staphyloferrin A (SA) and staphyloferrin B (SB) were administered as culture supernatants derived from an *S. aureus* Δ*sbn*Δ*sfa* mutant strain unable to produce siderophore as well as *in-vitro* synthesized siderophores. DFO; Deferoxamine. Data presented are the mean ± standard deviation from at least three independent experiments.**
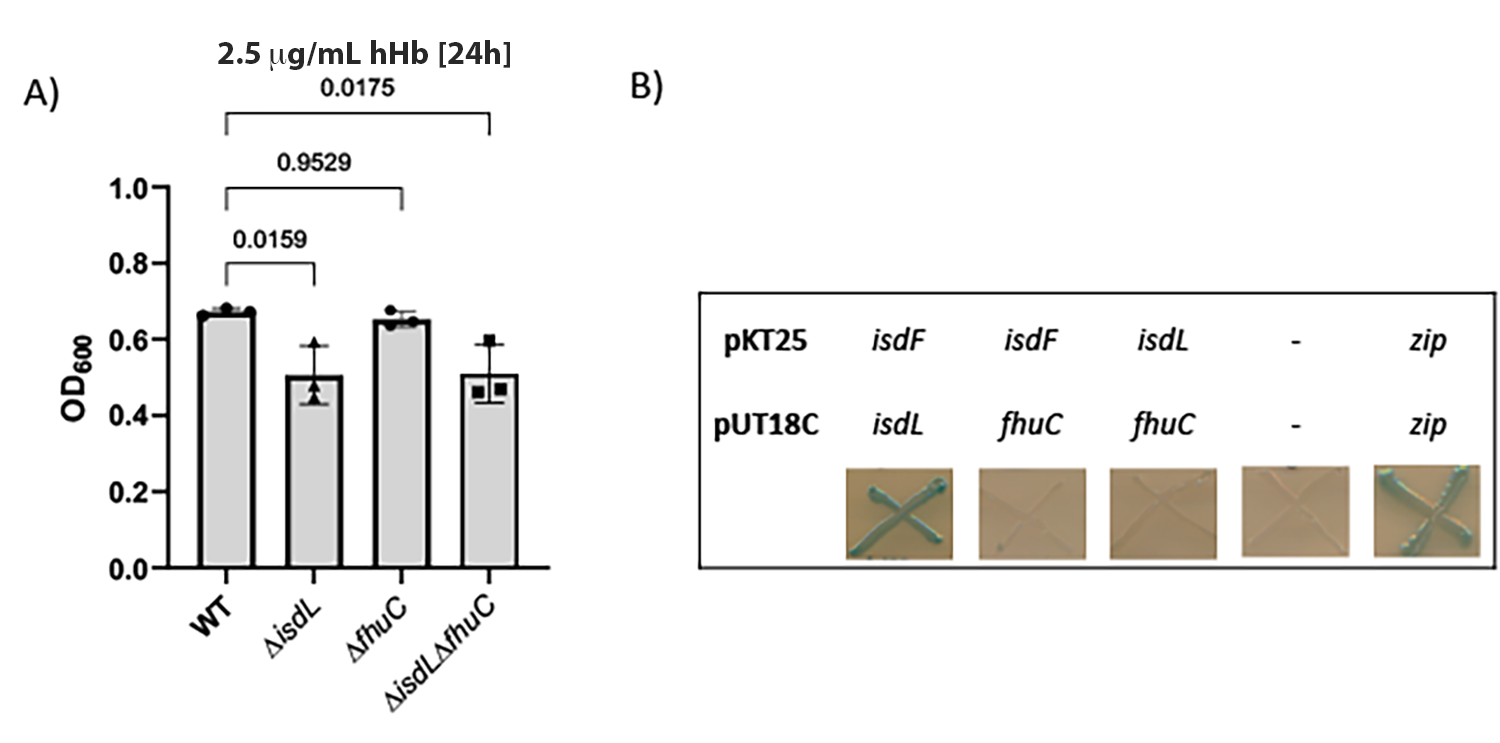
**

**Figure S4. IsdL-dependent heme acquisition from hemoglobin in *S. lugdunensis*.** In A) the growth of *S. lugdunensis* N920143 wildtype (WT) and ATPase deficient mutants Δ*isdL*, Δ*fhuC and* Δ*isdL*Δ*fhuC* is shown. Strains were grown in the presence of 2.5 µg/mL human hemoglobin (hHb). 500 µL of RPMI + 1% casamino acids + 10 µM EDDHA were inoculated to an OD_600_ of 0.005 in 48 well plates, OD_600_ was measured every 15 min in an Epoch2 plate reader. For reasons of clarity, values after 24 hrs are shown. Mean and SD of three experiments are displayed. Statistical analysis was performed using one-way ANOVA followed by Dunnett‘s test for multiple comparison. B) shows the BACTH assay (Euromedex) of the permease IsdF and the ATPases IsdL and FhuC (*S. lugdunensis* N920143). The proteins were cloned into pKT25 and pUT18C, respectively, and co-transformed into *E. coli* BTH101. As negative control empty vectors (-), as positive control leucine zippers (zip) were used. BTH101 strains were streaked onto LB agar containing X-Gal IPTG ampicillin and kanamycin and incubated for 2 days at 30°C. Blue colour indicates protein-protein interaction.

**
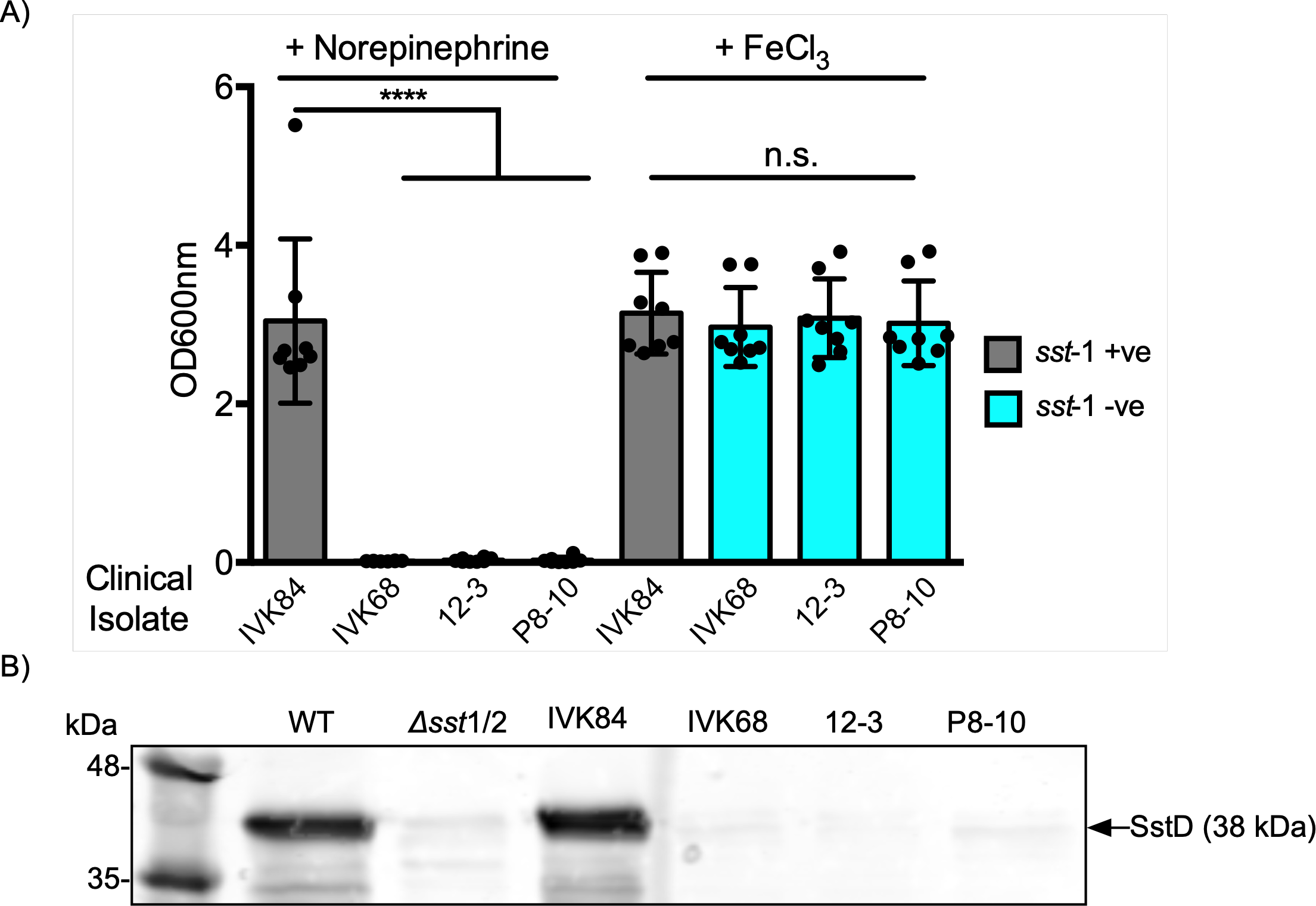
**

**Figure S5. Clinal isolates of *S. lugdunensis* lacking the sst-1 locus fail to utilize norepinephrine to support growth.** In (A), the ability of the identified *S. lugdunensis* clinical isolates to utilize norepinephrine (NE) as an iron source is shown. The bacteria were grown in RPMI with 1% (v/v) casamino acids and 0.1% (v/v) heat inactivated horse serum. NE was added at 50 μM and FeCl_3_ was used as a control at 20 μM. The data shown are the mean ± standard deviation of the endpoint optical density at 600nm (OD600nm) measured after 24 hr. The data derive from three independent experiments and each symbol represents a separate biological replicate. Statistical significance was determined by ordinary one-way ANOVA with a tukey’s post-test. In B and D, n.s. indicates not significant, *p<0.05, ****p<0.0001. In (B), representative western blot of whole cell lysates from indicated strains grown in RPMI + 1% CAs and probed with an anti-SstD antiserum. The SstD protein is indicated.


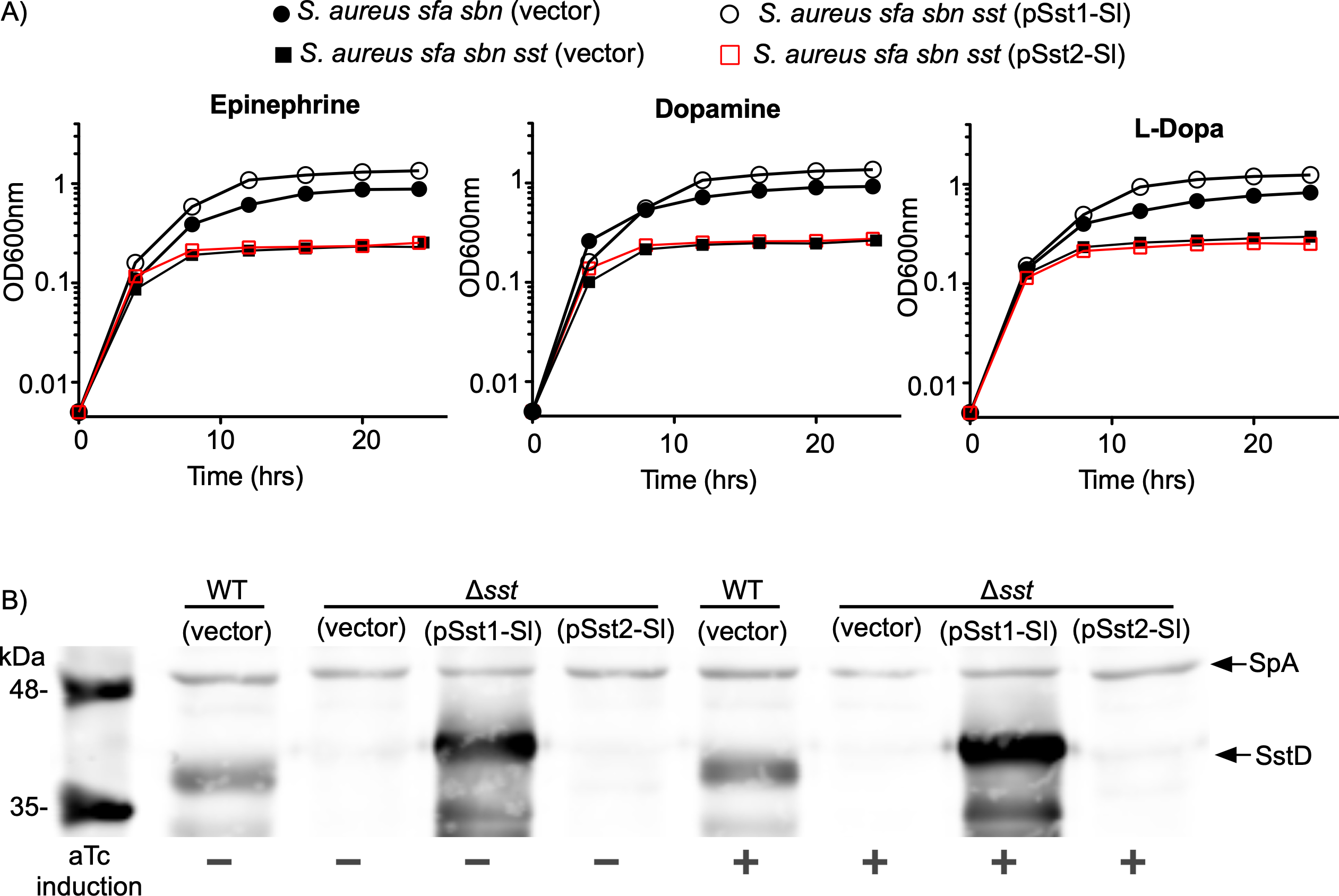


**Figure S6. The *sst*-1 locus of *S. lugdunensis* is required for use of host catecholamine stress hormones as iron sources.** Growth of *S. aureus sfa sbn sst* was analyzed in C-TMS with 20% serum, supplemented with 50μM of the indicated catecholamine hormones. *S. aureus* lacking *sfa* and *sbn* is labeled here as WT strain as the *sst* locus in this *S. aureus* background is intact. The strain listed as Δ*sst* carries a deletion of the *sst* locus in addition to the *sfa sbn* mutations. The bacteria were transformed with either vector control or the pSst-1 or pSst-2 plasmids that encode either the *sst*-1 or *sst*-2 locus from *S. lugdunensis*, respectively. Deferoxamine (DFO) was used as a positive control as all strains can transport the hydroxamate DFO. In (A) the data are the average of at least three independent biological replicates from three independent experiments. In (B) the western blot data show the expression of SstD by the indicated *S. aureus* strain Newman mutants carrying the indicated plasmids. Here, the *S. aureus* Newman *sfa sbn* mutant but encoding a functional *sst* locus is labeled as WT whereas Δ*sst* is *S. aureus* Newman lacking the *sfa sbn sst* loci. Lysates were derived from bacteria grown in RPMI + 1%(w/v) casamino acids supplemented with antibiotic and with or without aTc (250 ng/mL) to induce gene expression as indicated. The arrow labeled SpA points to the *S. aureus* protein A that is present in each sample and the arrow labeled SstD points to SstD expressed by WT *S. aureus* or the Δ*sst* strains carrying pSst1.

**
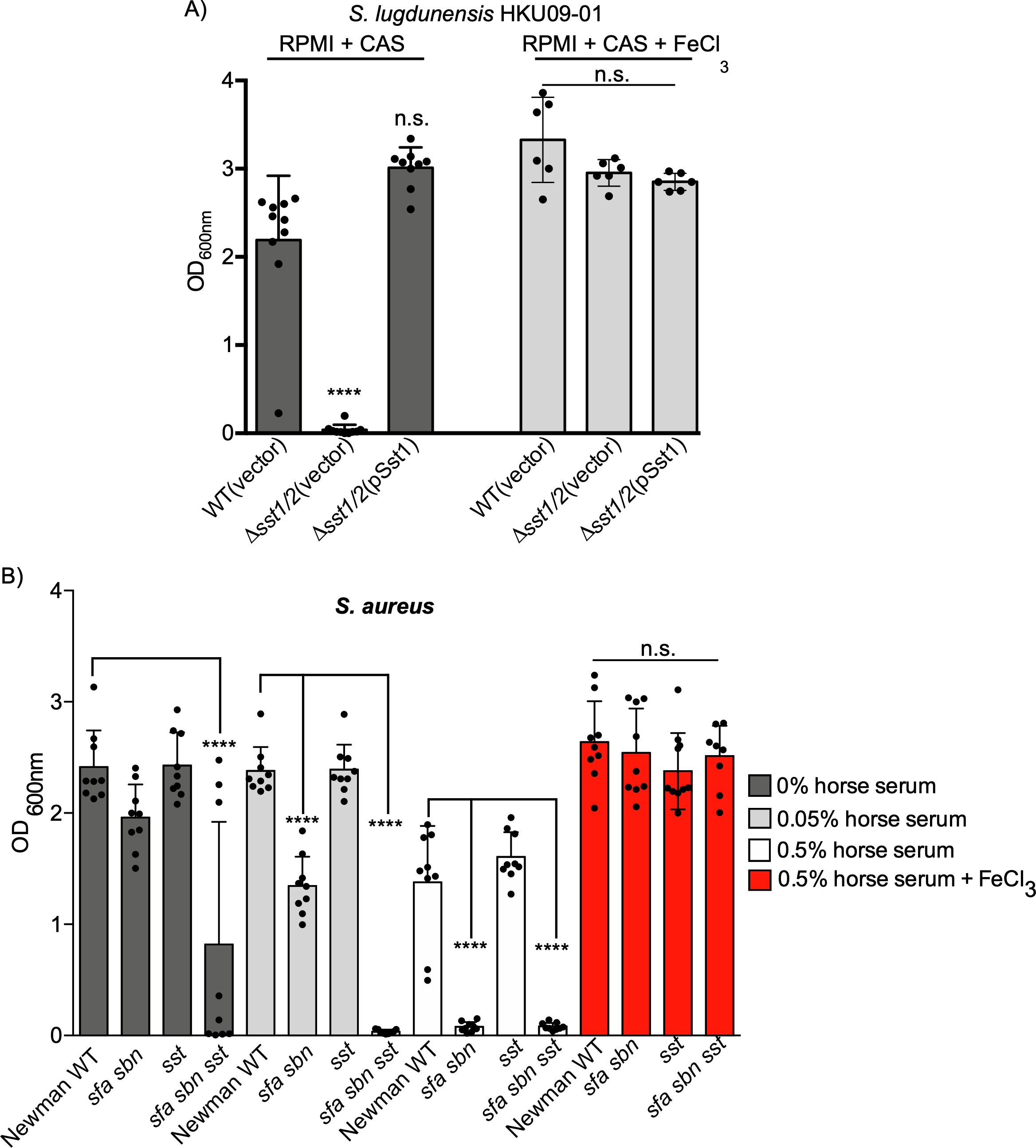
**

**Figure S7. Casamino acids contain factors that are utilized by *S. lugdunensis* and *S. aureus* as iron sources in an Sst-dependent manner**. In (A) growth of wildtype *S. lugdunensis* and the Δ*sst*-1/2 mutant in RPMI supplemented with 1% (w/v) casamino acids in the absence of any other chelator (i.e., horse serum) is shown. The data shown are the mean ± standard deviation of the measured OD600nm after 24h. Bacteria were pre-cultured in serum free RPMI overnight in the presence of antibiotics to maintain plasmid selection. The addition of FeCl_3_ was used as a control to show the inability of the Δ*sst*-1/2 mutant to grow is due to Fe starvation. Statistically significant differences in growth were determined using an ordinary one-way ANOVA with a Dunnett’s multiple comparison test using wildtype as a comparator. In (B) growth of *S. aureus* strain Newman and siderophore biosynthesis/utilization mutants in RPMI with 1% (w/v) casamino acids is shown. Bacteria were pre-cultured in serum free RPMI overnight to deplete the cells of Fe and then inoculated into RPMI + casamino acids in the presence of increasing concentration of horse serum. The data shown are the mean ± standard deviation of the measured OD600nm after 24h. Statistically significant differences in growth were determined using an ordinary one-way ANOVA with a Dunnett’s multiple comparison test using each wildtype *S. aureus* Newman at a given horse serum concentration as a comparator. In (A) and (B) n.s denotes not significant and ****p<0.0001.

**Table S1.** Bacterial strains, plasmids and oligonucleotides used in this study

| **Bacterial strain, plasmid or oligonucleotide** | **Description** | | **Source or reference** |  |
| --- | --- | --- | --- | --- |
| **Strains** |  | |  |  |
| ***E. coli*** |  |  |  |  |
| DH5α | Φf80dlacZΔM15 recA1 endA1 gyrAB thi-1 hsdR17(r_K_^−^ m_K_^−^) supE44 relA1 deoR *Δ(lacZYA-argF)U169* | | Promega |  |
| SL01B | Expresses the *hsdMS* from *S. lugdunensis* N920143 (CC1) | | (1) |  |
| BL21λ(DE3) | F^−^ompThsdS_B_(r_B_^−^ m_B_^−^) dcmgal λ(DE3) | | Novagen |  |
| H3320 | BL21λ(DE3) pET28::*sst1D*; Km^R^ | | This study |  |
| H3321 | BL21λ(DE3) pET28::*sst2D*; Km^R^ | | This study |  |
| BTH101 | Used for BACTH assay  F-, *cya-99*, *araD139, galE15, galK16, rpsL1 (Str r)*, *hsdR2, mcrA1, mcrB1* | | BACTH System  (Euromedex) |  |
|  | BTH101 pKT25:*isdF* + pUT18C:*isdL* | | This study |  |
|  | BTH101 pKT25:*isdF* + pUT18C:*fhuC* | | This study |  |
|  | BTH101 pKT25 + pUT18C | | This study |  |
|  | BTH101 pKT25:*zip* + pUT18C:*zip* | | This study |  |
| ***S. lugdunensis*** |  |  |  |  |
| HKU09-01 | Human skin infection isolate | | (2) |  |
| N920143 | Human abscess | | (3) |  |
| H2710 | HKU09-01 Δ*isd*-*sir* | | (4) |  |
| H2970 | HKU09-01 Δ*fhuC* | | This study |  |
| H3016 | HKU09-01 Δ*sst* | | This study |  |
| H3325 | HKU09-01 Δ*isd*-*sir* Δ*fhuC* Δ*sst* | | This study |  |
| H3316 | HKU09-01 Δ*fhuC* pRMC2 empty vector control; Cm^R^ | | This study |  |
| H3317 | HKU09-01 Δ*fhuC* (pfhuC); Cm^R^ | | This study |  |
| H3883 | HKU09-01 Δ*feoAB* | | This study |  |
| H3884 | HKU09-01 Δ*fhuC* Δ*sst* Δ*feoAB* | | This study |  |
| H3887 | HKU09-01 Δ*sitABC* Δ*feoAB* | | This study |  |
| H3894 | HKU09-01 Δ*fhuC* Δ*sst* Δ*sitABC* | | This study |  |
| H3890 | HKU09-01 Δ*fhuC* Δ*sst* Δ*sitABC* Δ*feoAB* | | This study |  |
| H3895 | HKU09-01 Δ*isd*-*sir* Δ*fhuC* Δ*sst* Δ*sitABC* Δ*feoAB* | | This study |  |
| H4056 | HKU09-01 pALC2073 empty vector control; Cm^R^ | | This study |  |
| H4295 | HKU09-01 Δ*fhuC* Δ*sst* Δ*feoAB* (pALC2073); Cm^R^ | | This Study |  |
| H4296 | HKU09-01 Δ*fhuC* Δ*sst* Δ*feoAB* (pFeoAB) | | This study |  |
| H4297 | HKU09-01 Δ*fhuC* Δ*sst* Δ*sitABC* Δ*feoAB* (pALC2073); Cm^R^ | | This study |  |
| H4298 | HKU09-01 Δ*fhuC* Δ*sst* Δ*sitABC* Δ*feoAB* (pFeoAB); Cm^R^ | | This study |  |
|  | N920143 Δ*isdL* | | This study |  |
|  | N920143 Δ*fhuC* | | This study |  |
| **Clinical Isolates** |  | |  |  |
| 84 | Nasal isolate, sst-1 positive, sst-2 positive | | This study |  |
| 12-3 | Nasal isolate, sst-1 negative, sst-2 positive | | This study |  |
| 68 | Nasal isolate, sst-1 negative, sst-2 positive | | This study |  |
| SL4 | Nasal isolate, sst-1 negative, sst-2 positive | | This study |  |
| ***S. aureus*** |  |  |  |  |
| RN4220 | Prophage-cured laboratory strain; r_K_^−^ m_K_^+^; accepts foreign DNA | | (5) |  |
| H1666 | Newman ΔsbnABCDEFGHI::Tc ΔsfaABCsfaD::Km; Tc^R^ Km^R^ | | (6) |  |
| H2224 | Newman ΔsstABCD::Em ΔsbnABCDEFGHI::Tc ΔsfaABCsfaD::Km; Em^R^ Tc^R^ Km^R^ | | (6) |  |
| H3311 | H1666 pRMC empty vector control; Tc^R^ Km^R^ Cm^R^ | | This study |  |
| H3312 | H2224 pRMC empty vector control; Em^R^ Tc^R^ Km^R^ Cm^R^ | | This study |  |
| H3313 | H2224 pRMC::*sst1; sst* mutant complemented with *S.lugdunensis sst1ABCD*; Em^R^ Tc^R^ Km^R^ Cm^R^ | | This study |  |
| H3314 | H2224 pRMC::*sst2; sst* mutant complemented with *S.lugdunensis sst2ABCD*; Em^R^ Tc^R^ Km^R^ Cm^R^ | | This study |  |
| H2508 | USA300 LAC cured of its endogenous resistance plasmid | | Lab stock |  |
| **Plasmids** |  |  |  |  |
| pKOR1 | *E. coli/Staphylococcus* shuttle vector allowing allelic replacement in staphylococci; Ap^R^ Cm^R^ | | (7) |  |
| pKOR1Δ*fhuC* | pKOR1 plasmid for in-frame deletion of *fhuC*; Ap^R^ Cm^R^ | | This study |  |
| pKOR1Δ*sst* | pKOR1 plasmid for deletion of genetic region encompassing duplicated *sstABCD*; Ap^R^ Cm^R^ | | This study |  |
| pKORΔ*feoAB* | pKOR1 plasmid for in-frame deletion of *feoAB*; Ap^R^ Cm^R^ | | This Study |  |
| pKORΔ*sitABC* | pKOR1 plasmid for in-frame deletion of *sitABC*; Ap^R^ Cm^R^ | | This Study |  |
| pRMC2 | *E. coli/Staphylococcus* shuttle vector: Ap^R^ Cm^R^ | | (8) |  |
| pALC2073 | *E. coli/Staphylococcus* shuttle vector: Ap^R^ Cm^R^ | | (9) |  |
| pFhuC | pRMC2 derivative for *fhuC* expression; Ap^R^ Cm^R^ | | This study |  |
| pSst1 | pRMC2 derivative for *sst1ABCD* expression; Ap^R^ Cm^R^ | | This study |  |
| pSst2 | pRMC2 derivative for *sst2ABCD* expression; Ap^R^ Cm^R^ | | This study |  |
| pFeoAB | pALC2073 derivative carrying *feoAB*; Ap^R^ Cm^R^ | | This Study |  |
| pET28a(+) | *E. coli* vector for overexpression of recombinant hexahistidine-tagged proteins; Km^R^ | | Novagen |  |
| pET28::*sst1D* | pET28a(+) derivative encoding N-terminally hexahistidine-tagged soluble portion of Sst1D; Km^R^ | | This study |  |
| pET28::*sst2D* | pET28a(+) derivative encoding N-terminally hexahistidine-tagged soluble portion of Sst2D; Km^R^ | | This study |  |
| pIMAY | *E. coli/Staphylococcus* shuttle vector allowing allelic replacement in staphylococci; Ap^R^ Cm^R^ | | (10) |  |
| pIMAY:Δ*isdL* | pIMAY plasmid for in-frame deletion of *isdL*; Ap^R^ Cm^R^ | | This study |  |
| pIMAY:Δ*fhuC* | pIMAY plasmid for in-frame deletion of *fhuC*; Ap^R^ Cm^R^ | | This study |  |
| pKT25 | BACTH assay plasmid, N-terminal T25 fragment;Km^R^ | | BACTH System  (Euromedex) |  |
| pKT25:*isdF* | T25 fragment N-terminally of *isdF*; Km^R^ | | This study |  |
| pKT25:*zip* | Positive control plasmid, T25 fragment N-terminally of *zip* (leucine zipper); Km^R^ | | BACTH System  (Euromedex) |  |
| pUT18C | BACTH assay plasmid, N-terminal T18 fragment; Ap^R^ | | BACTH System  (Euromedex) |  |
| pUT18C:*isdL* | T18 fragment N-terminally of *isdL*; Ap^R^ | | This study |  |
| pUT18C:*fhuC* | T18 fragment N-terminally of *fhuC*; Ap^R^ | | This study |  |
| pUT18C:*zip* | Positive control plasmid, T18 fragment N-terminally of *zip* (leucine zipper); Ap^R^ | | BACTH System  (Euromedex) |  |
| **Oligonucleotides^b,c^** |  |  |  |  |
| Purpose | Nucleotide Sequence (5'-3') | | | |
| Primers for generating upstream and downstream recombinant regions for Δ*fhuC* using pKOR1 | | **(*AttB1*)-fhuCUF:***GGGGACAAGTTTGTACAAAAAAGCAGGCT* CTTGGTATTGGGATAATCG  **fhuCUR:** GTTGTCCATTCAAGCGAC  **fhuCDF:**Phos/CAGGCAAACCATTATTAGTTACC  **(AttB2)-fhuCDR:***GGGGACCACTTTGTACAAGAAAGCTGGGT* TGTCAATGGCAATACTTTAG | | |
| Primers for generating upstream and downstream recombinant regions for Δ*sst* using pKOR1 | | **(*AttB1*)sstUF:***GGGGACAAGTTTGTACAAAAAAGCAGGCT*TATTGCTCGGGATCAAG  **sstUR:** GCCAACAAACAATGAAATG  **sstDF:** Phos/AAATCATCAGCCAAACAGG  **(*AttB2*)-sstDR:***GGGGACCACTTTGTACAAGAAAGCTGGGT*AAACACGCTGG  CTTTATG | | |
| Primers for generating upstream and downstream recombinant regions for Δ*feoAB* using pKOR1 | | **(AttB1)-feoUF:***GGGGACAAGTTTGTACAAAAAAGCAGGCT*CGATAAAGAA  GTGCCTAAGTG  **feoUR:** GGACCTCCGCGGTACACCACTCCAGTTACCTAC  **feoDF:** GGACCTCCGCGGAGTTCATGGAAATGGACACTCAT  **AttB2-feoDR:***GGGGACCACTTTGTACAAGAAAGCTGGGT*TACTGCAAGCAT  TGATTTGGG  **Feo-F:** CAGCAGAATGGTTGAAAAAAGG  **FeoR:** AGTAAAACGACCGATGACATAC | | |
| Primers for generating upstream and downstream recombinant regions for Δ*sitABC* using pKOR1 | | **(AttB1)-sitUF:***GGGGACAAGTTTGTACAAAAAAGCAGGCT*TCAAATTCTGAC  TTCTTGCTCC  **sitUR:**GGACCTCCGCGGATCTTTCAAGACAATTTTACCGT  **sitDF:**GGACCTCCGCGGGGTACACCTGAACAAATGAAAC  **(AttB2)sitDR:***GGGGACCACTTTGTACAAGAAAGCTGGGT*GTTTGGGGAGTGTATGGGTT  **Sit-F:** TAACTACCCCCATCTTATAGCTT  **Sit-R:** CTGCATTAAAAATTAGAGAAGCGA | | |
| Primers for cloning *fhuC* into pRMC2 for complementation | | **KpnI-fhuCF:** GATCGGTACCAAGACGCAAGTGTCAAGAG  **SacI-fhuCR:** GATCGAGCTCACAGCACCTAAATCTCTTGG | | |
| Primers for cloning *sst1* into pRMC2 for complementation | | **KpnI-sst1F**: GATCGGTACCTGCCTTAGACACAACGAC  **SacI-sst1R**: GATCGAGCTCGACTCGTAAGAAAGCAAACC | | |
| Primers for cloning *sst2* into pRMC2 for complementation | | **EcoRI-sst2F**: GATCGAATTCAGGTTCTGTTGTTGGTGG  **EcoRI-sst2R**: GATCGAATTCTAAATGTTGTCCCGCTCC | | |
| Primers for cloning *sst1D* into pET28a(+) for overexpression | | **NdeI-sst1DF:** GATCCATATGGAAACAAAGAGTGGCGAATCA  **SacI-sst1DR:** GATCGAGCTCGGAATGATATCCCCACTTCA | | |
| Primers for cloning *sst2D* into pET28a(+) for overexpression | | **NdeI-sst2DF:** GATCCATATGAGCTCAGATGCTAAGTCATCA  **SacI-sst2DR:** GATCGAGCTCGCTAAACAAGATGTCTTGAAAT | | |
| Primers for cloning *feoAB* | | **F:**TATATAGGTACCGAAAGAGTTATAATACGAATTTAAG  **R:**TATATAGAGCTCCGTTCATTCAAAAATAGAAAAGC | | |
| Primers for RT-PCR of *rpoB* | | **F:** AGAGAAAGACGGCACTGAAAACAC  **R:** ATAACGACCCACGCTTGCTAAG | | |
| Primers for RT-PCR of *fhuC* | | **F:** TGGACCAAATGGATGTGG  **R:** GCTACTTCTGGAGATTGTGG | | |
| Primers for RT-PCR of *sst1A* | | **F:** CTCGTTTGCTTTCCTCAAG  **R:** TGCCACCCAACATAATACC | | |
| Primers for RT-PCR of *sst2A* | | **F:** GGCATTATGTTAGGTGGTATTG  **R:** CGTCCACTTGTAATAATGGC | | |
| Primers for generating upstream and downstream recombinant regions for N920143 Δ*fhuC* using pIMAY | | **ΔfhuC_PF-A_SacI**: GAATGGagcTCTTGTCGAAGAATAATCAAAG  **ΔfhuC_PR-B:** CATAATTCCCCAACTTTCTATTTATTCTC  **ΔfhuC_PF-C:** ATAGAAAGTTGGGGAATTATGTAAAGATATTTTGAAAAGGATACG  **ΔfhuC_PR-D_KpnI:** AGAATggTaCcCATGGGATTAAATCCGTCAC  **ΔfhuC_Sc.F:** TGTACGTGGTCATCAGTAAGACGCAAG  **ΔfhuC_Sc.R:** GACCAAAACGTCAACGATTGATTTAATC | | |
| Primers for generating upstream and downstream recombinant regions for N920143 Δ*isdL* using pIMAY | | **ΔisdL_PE:**  AGGGAACAAAAGCTGGGTACCGTTAAATGGCATACTAGAACTG  **ΔisdL_PF:**  TAATCGAAGCACTAATTTCACATGACTTACCGCTCCTTTACA  **ΔisdL_C:**  TGAAATTAGTGCTTCGATTATG  **ΔisdL_D:**  CTATAGGGCGAATTGGAGCTCGATATTTTGTATCGAATTGAATGC  **IsdL_Sc.F:**  AATATAAATTAGCGCCAGTGAG  **IsdL_Sc.R:** CTTTCGTCGTTGTTTGATAAGC | | |
| Primers for generating plasmids for BACTH assay | | **pKT25:*isdF***  **PF_IsdF_KT_PstI:**  AAGAGGctGcaGATATGAAAAAACATCCAGC  **PR_IsdF_all_SmaI_nostop:**  TCGTAcccgggGAGATGTTCTATGCGCATG  **pUT18C:*isdL***  **PF_IsdL_KNT,18,18C_PstI:** AGGActGcagAGTCATGCGCATAGAACATCTTAAC  **PR_IsdL_all_SmaI_nostop:** GAAGCcCcgggTTTTGTTGTGTCCGCCTCG  **pUT18C:*fhuC***  **PF_FhuC_KNT,18,18C_PstI:** AGTTGctGcAgTATGAGTCGCTTGAATGGAC  **PR_FhuC_all_SmaI_nostop:** TTTCAACCCGggTTGAGTATGTTTTACTAAAC | | |

1. Ap^R^, Cm^R^, Km^R^, Em^R^ and Tc^R^; resistance to ampicillin, chloramphenicol, kanamycin,
    erythromycin and tetracycline, respectively.
2. Restriction sites for cloning are underlined, AttB1/2 sites are italicized
3. Phos/ denotes a 5' phosphate on the primer.

**Supporting information references**

1. Heilbronner, S., Hanses, F., Monk, I. R., Speziale, P., and Foster, T. J. (2013) Sortase A promotes virulence in experimental *Staphylococcus lugdunensis* endocarditis. *Microbiology*. **159**, 2141–2152

2. Tse, H., Tsoi, H. W., Leung, S. P., Lau, S. K. P., Woo, P. C. Y., and Yuen, K. Y. (2010) Complete genome sequence of *Staphylococcus lugdunensis* strain HKU09-01. *J. Bacteriol.* **192**, 1471–1472

3. Heilbronner, S., Holden, M. T. G., van Tonder, A., Geoghegan, J. A., Foster, T. J., Parkhill, J., and Bentley, S. D. (2011) Genome sequence of *Staphylococcus lugdunensis* N920143 allows identification of putative colonization and virulence factors. *FEMS Microbiol. Lett.* **322**, 60–67

4. Brozyna, J. R., Sheldon, J. R., and Heinrichs, D. E. (2014) Growth promotion of the opportunistic human pathogen, *Staphylococcus lugdunensis*, by heme, hemoglobin, and coculture with *Staphylococcus aureus*. *Microbiologyopen*. **3**, 182–195

5. Kreiswirth, B. N., Löfdahl, S., Betley, M. J., O’Reilly, M., Schlievert, P. M., Bergdoll, M. S., and Novick, R. P. (1983) The toxic shock syndrome exotoxin structural gene is not detectably transmitted by a prophage. *Nature*. **305**, 709–712

6. Beasley, F. C., Marolda, C. L., Cheung, J., Buac, S., and Heinrichs, D. E. (2011) Staphylococcus aureus transporters Hts, Sir, and Sst capture iron liberated from human transferrin by Staphyloferrin A, Staphyloferrin B, and catecholamine stress hormones, respectively, and contribute to virulence. *Infect. Immun.* **79**, 2345–55

7. Bae, T., and Schneewind, O. (2006) Allelic replacement in *Staphylococcus aureus* with inducible counter-selection. *Plasmid*. **55**, 58–63

8. Corrigan, R. M., and Foster, T. J. (2009) An improved tetracycline-inducible expression vector for *Staphylococcus aureus*. *Plasmid*. **61**, 126–129

9. Bateman, B. T., Donegan, N. P., Jarry, T. M., Palma, M., and Cheung, a L. (2001) Evaluation of a tetracycline-inducible promoter in *Staphylococcus aureus* in vitro and in vivo and its application in demonstrating the role of sigB in microcolony formation. *Infect. Immun.* **69**, 7851–7857

10. Monk, I. R., Shah, I. M., Xu, M., Tan, M. W., and Foster, T. J. (2012) Transforming the untransformable: Application of direct transformation to manipulate genetically *Staphylococcus aureus* and *Staphylococcus epidermidis*. *MBio*. **3**, 1–11
